# Host-microbe multi-omics and succinotype profiling have prognostic value for future relapse in patients with inflammatory bowel disease

**DOI:** 10.1080/19490976.2025.2450207

**Published:** 2025-01-15

**Authors:** Jill O’Sullivan, Shriram Patel, Gabriel E. Leventhal, Rachel S. Fitzgerald, Emilio J. Laserna-Mendieta, Chloe E. Huseyin, Nina Konstantinidou, Erica Rutherford, Aonghus Lavelle, Karim Dabbagh, Todd Z. DeSantis, Fergus Shanahan, Andriy Temko, Shoko Iwai, Marcus J. Claesson

**Affiliations:** a School of Microbiology, University College Cork, Cork, Ireland; b APC Microbiome Ireland, University College Cork, Cork, Ireland; c SFI Centre for Research Training in Genomics Data Science, University of Galway, Galway, Ireland; d SeqBiome Ltd, Cork, Ireland; e PharmaBiome AG, Schlieren, Zurich, Switzerland; f Department of Informatics, Second Genome Inc, South San Francisco, California, USA; g Department of Anatomy and Neuroscience, University College Cork, Cork, County Cork, Ireland; h Department of Medicine, University College Cork, Cork, Ireland; i Department of Electrical and Electronic Engineering, University College Cork, Cork, Ireland

**Keywords:** Crohn’s disease, ulcerative colitis, inflammatory bowel disease, gut microbiome, host-microbe interactions, machine learning

## Abstract

Crohn’s disease (CD) and ulcerative colitis (UC) are chronic relapsing inflammatory bowel disorders (IBD), the pathogenesis of which is uncertain but includes genetic susceptibility factors, immune-mediated tissue injury and environmental influences, most of which appear to act via the gut microbiome. We hypothesized that host-microbe alterations could be used to prognostically stratify patients experiencing relapses up to four years after endoscopy. We therefore examined multiple omics data, including published and new datasets, generated from paired inflamed and non-inflamed mucosal biopsies from 142 patients with IBD (54 CD; 88 UC) and from 34 control (non-diseased) biopsies. The relapse-predictive potential of 16S rRNA gene and transcript amplicons (standing and active microbiota) were investigated along with host transcriptomics, epigenomics and genetics. While standard single-omics analysis could not distinguish between patients who relapsed and those that remained in remission within four years of colonoscopy, we did find an association between the number of flares and a patient’s succinotype. Our multi-omics machine learning approach was also able to predict relapse when combining features from the microbiome and human host. Therefore multi-omics, rather than single omics, better predicts relapse within 4 years of colonoscopy, while a patient’s succinotype is associated with a higher frequency of relapses.

## Introduction

The microbiome has been implicated in the pathogenesis of Crohn’s disease and ulcerative colitis, collectively described as inflammatory bowel disease (IBD). Although alterations to the gut microbiome composition have been reported prior to the onset of clinically overt disease in subjects at increased risk of developing IBD by us and others,^1–7^ many of the microbiome compositional anomalies are linked with and may be secondary to the presence of inflammation. Thus, we previously found disturbances in the microbiome in a cross-sectional study of biopsies from inflamed and non-inflamed segments of the bowel in both forms of IBD.^5,8^ Moreover, in a separate longitudinal study, we showed that fecal microbiome disturbances were associated with active disease rather than remission.^4^

While an undisputed link exists between the microbiome and IBD, this is just one of multiple factors involved in the disease.^9^ Therefore, multi-omics analysis of both host and microbiome data offers novel opportunities to further our understanding of these chronic disorders. We previously showed that by combining microbiota data with host features, it was possible to improve the classification of disease and inflammation status of samples from patients with IBD.^5^ Priya and coauthors also found associations between microbiota and host gene expression profiles, several of which were specific to IBD when compared to other gastrointestinal disorders,^10^ while another study identified context-specific mucosal host-microbe interactions within patients with IBD.^11^

While the number of IBD studies using multi-omics data is increasing, few have applied these datasets to predict future disease outcomes in patients. Of those that have, the focus has been on multi-omics data from either the host^12^ or the microbiome,^13^ rather than the integration of both. Here, we assess disease outcome four years after initial microbiome sampling^5^ to determine if a multi-omics profile consisting of both microbiome and host data might be of value in prognostically stratifying patients. The results suggest that a multi-omics strategy (rather than single omics) is more predictive of relapse four years after colonoscopy while a patient’s succinotype is associated with a higher frequency of relapses.

## Materials and methods

The subjects included in this study were recruited as described previously.^5^ Briefly, these subjects were all undergoing colonoscopy or sigmoidoscopy as part of their ongoing care and volunteered to provide biopsy material for research. For patients with CD, colonic biopsies were collected from inflamed and non-inflamed regions. In the case of UC subjects, biopsies were taken from the distal inflamed and proximal non-inflamed segment of the colon. Those controls included in the study consisted of subjects undergoing colonoscopy for cancer or other disease screening in which no significant colonic or gastrointestinal disorder was found.

## Data generation

### Nucleic acid extraction and sequence data generation

Biopsies were completely defrosted in RNA-later before performing DNA/RNA purification with AllPrep DNA/RNA/Protein Mini kit (Qiagen). Defrosted biopsies were transferred into a tube containing 350 µL RLT buffer with β-mercaptoethanol (Sigma-Aldrich, St Louis, MO), three 3.5 mm glass beads and 0.25 mL of 0.1 mm glass beads (Biospec, Bartlesville, OK). Disruption and homogenization were carried out in a MagNA Lyser (Roche, Penzberg, Germany) twice for 15 seconds at 3,500 or 6,500 rpm. PERMANOVA test confirmed the different centrifugation speed did not significantly affect microbiota (data not shown). Subsequent DNA/RNA purification was performed according to the kit manufacturer’s instructions. DNA contaminations in RNA samples were removed by Turbo DNA-free kit following manufacturer’s instructions (Ambion, Carlsbad, CA). DNA and RNA concentrations were measured using a Nano-Drop 2000 Spectrophotometer (Thermo Scientific, Waltham, MA). DNA and RNA integrity were checked on 1% agarose gel electrophoresis and 2100 Bioanalyzer system (Agilent Technologies, Santa Clara, CA), respectively. In addition, RNA quality was considered acceptable if RNA integrity number ≥ 6 and rRNA ratio ≥ 1.5. Nucleic acid extracts were stored at − 80°C until further downstream applications. For 16S cDNA analysis, total RNA was reverse transcribed using High Capacity cDNA Reverse Transcription kit following manufacturer’s instructions (Applied Biosystems, Foster City, CA). The PCR was employed to amplify 16S rRNA V3-V4 hypervariable region using 341F and 805 R primer set with Nextera transposase adaptors^14^: 16S_V3_341F, TCGTCGGCAGCGTCAGATGTGTATAAGAGACAGCCTACGGGNGGCWGCAG; 16S_V4_805R, GTCTCGTGGGCTCGGAGATGTGTATAAGAGACAGGACTACHVGGGTATCTAATCC. Template DNA or cDNA was mixed with primers at a concentration of 0.2 μM and Phusion High-Fidelity DNA Polymerase for a total volume of 30 μL (Thermo Scientific). PCR conditions were 98°C for 30 sec, 30 cycles of 98°C for 10 sec, 55°C for 15 sec and 72°C for 20 sec, with final elongation at 72°C for 5 minutes. PCR products were verified with a presence of a band on an agarose gel and purified using Agencourt AMPure XP magnetic beads (Beckman-Coulter, Brea, CA). Purified DNA product was eluted in 50 μL EB buffer (Qiagen). Using 5 μL of the PCR products as template, eight additional cycles of PCR was conducted with Illumina primers containing Nextera XT indexes (Illumina, San Diego, CA) and Phusion High-Fidelity DNA Polymerase in a final volume of 50 μL, then purified using Agencourt AMPure XP magnetic beads. The amplicon concentrations were measured using Quant-iT Picogreen dsDNA assay kit (Thermo Scientific). Libraries were pooled equimolar and sequenced by Illumina MiSeq for 2 × 300bp reads at Eurofins Genomics (Ebersberg, Germany).

The quality of raw reads was visualized using FastQC v0.11.5^15^ followed by first-pass quality filtering using Trimmomatic v0.39^16^ with the following parameters: SLIDINGWINDOW:5:20 AVGQUAL:20 minLEN:200. A big data pipeline was used to infer Ribosomal Sequence Variants (RSVs) using the DADA2 v1.20 considering the following parameters: truncLen=c(265,220), trimLeft=c(17,21), maxEE=c(2,2), truncQ=c(2,2), maxN = 0, rm.phix=TRUE.^16^ We carried out error correction for the samples sequenced across multiple sequencing runs separately until chimera removal step as the error rates might differ between runs. Resulting non-chimeric RSVs were again chimera filtered using reference-based chimera filtering implemented in USEARCH v11^17^ with the ChimeraSlayer Gold database v2011051967. Taxonomy was assigned to non-chimeric sequences using assignTaxonomy function using SILVA database v138 with a bootstrap confidence threshold of 80%.^18^ Additionally, we used SPINGO for species level classification with the same reference database whenever possible.^19^

Initial pre-processing of 16S RSV table was conducted using the CoDaSeq package,^20^ whereby rare RSVs present in less than 5% of the samples in each of the datasets (gDNA biopsy, cDNA biopsy and stool) were removed using the codaseq.filter function. Overall, a total of 520 RSVs were retained with 318 RSVs from the gDNA biopsy, 435 RSVs from the cDNA biopsy and 361 RSVs from stool dataset. Except in the case of alpha diversity, this filtered RSV count table was used for all the downstream bioinformatic analysis.

Host RNA-Seq data was generated from mucosal biopsy samples as described previously.^5^ Trimmomatic v0.39 was used to trim adapters and remove low quality reads.^21^ Reads were quality checked using FastQC and multiQC before and after trimming. The quality filtered reads were then aligned to the human genome (GRCh38) using Hisat2 v2.1.0.^22^ A count table was then generated using featureCounts v1.5.0 using default parameters.^23^

### Host epigenome

Epigenetic data for CD samples (37 inflamed and 37 non-inflamed) and control samples (*n* = 22) was generated using the Illumina Infinium HumanMethylation 450 BeadChip array as described previously.^5^ In addition to this, epigenetic data is now also available for UC samples (30 inflamed and 46 non-inflamed) and some additional control samples (*n* = 14; 13/14 generated on both arrays) that were assayed using the Illumina Infinium HumanMethylation EPIC array. For both datasets, pre-processing and quality control was implemented using R libraries *minfi and minifiData*. Beta values were extracted and filtered using *BMIQ*^24^ for normalization between probe types with R libraries *methylumi* and *wateRmelon*. Probes were removed if the probe sequence mapped to multiple positions in the genome, mapped to sex chromosomes, had missing data or mapped to SNPs.

### Host genotype

Genotyping was carried out as described in Ryan et al.^5^ Due to the limited sample size, GWAS was not possible. Like in our previous study, we included 264 loci in our analysis which had been previously associated with IBD. This dataset was only considered for machine learning analysis.

### 16S G4 phylochip data

Libraries were prepared as previously described.^25^ Briefly, full-length V1-V9 16S rRNA genes were amplified from extracted DNA and purified. Approximately, 3.0 × 10^10^ double-stranded DNA molecules (500ng) from each library were combined with non-16S DNA spike-in controls, digested, and labeled. Each library was denatured and hybridized to its own G4 PhyloChip (non-multiplexed) in a 96-well high-throughput format, fluorescently stained and then scanned in a GeneTitan MC using GeneTitan Hybridization, Wash and Stain Kit for WT Array Plates (Thermo Fisher, Santa Clara, CA). The G4 PhyloChip queries 610,038 different 16S rRNA loci where a locus is defined as a 25-mer nucleotide sequence within a 16S rRNA gene within any of the 10 conserved regions or any of the 9 variable regions. Standard Affymetrix software (GeneChip Microarray Analysis Suite, ThermoFisher Scientific) was used to capture the scans of the array. Only perfect-match probes with fluorescence intensity observed in at least three samples were exported for rank-normalization in Sinfonietta software^26^ (Second Genome Inc, South San Francisco, CA) and were used as input to empirical probe-set discovery. All probe sets contained three or more probes and the empirical Operational Taxonomic Units (eOTU) tracked by a probe set were taxonomically annotated using StrainSelect version 2016.^27^ Analyses were conducted on hybridization scores (HybScores), which are the mean normalized rank for all probes within an eOTU. The probes were ranked according to their scaled-background subtracted fluorescence intensities. A total of 502 eOTUs were included in this analysis.

## Bioinformatics analysis

### Microbiome data

Statistical analysis and visualizations were performed in R v4.0 using the packages vegan,^28^ zCompositions,^29^ Tidyverse,^30^ rstatix,^31^ EnhancedVolcano^32^ and ggplot2.^33^ Due to the complex compositional nature of the microbiome data, we applied a centered log-ratio transformation (CLR) to each sample in our dataset. Datasets, such as 16S rRNA sequencing data, which are generated by next-generation sequencing (NGS) technologies are inherently compositional as the total number of reads produced are limited to the sequencing depth.^20^ As a result, many standard statistical approaches may not be appropriate as the independence assumption between features does not hold. The CLR transformation has, thus, been suggested as a suitable approach when conducting compositional data analysis (CoDA) as it compares log-ratios rather than raw sequencing counts.^20^ We first imputed the zeros in the abundance matrices using a count zero multiplicative replacement method (cmultRepl, method = “CZM”) implemented in the zCompositions package. Following this, we applied the CLR transformation using the codaSeq.clr function from the CoDaSeq package. The CLR transformation was applied separately to each taxonomic level in the RSV table (from phylum to species level). The Shannon diversity index was used to estimate the species richness and evenness and Wilcoxon test was used to evaluate statistical significance between clinical variables (e.g., disease type, biopsy type and future relapse). Paired testing was performed whenever possible. For beta diversity analysis, we used compositionally coherent Aitchison distance matrix and applied pairwise Permutational Multivariate Analysis of Variance (PERMAOVA) with 9,999 permutations to quantify community level differences.

Differential abundant taxa and genes were identified using ALDEx2 (ANOVA-Like Differential Expression), a compositionally-robust differential abundance analysis approach. ALDEx2 estimates per-feature technical variation within each sample using Monte-Carlo instances (*n* = 512) drawn from the Dirichlet distribution.^34^ This distribution maintains the proportional nature of the data.^20^ ALDEx2 uses the Centred Log-Ratio (CLR) transformation that ensures the data are scale invariant and sub-compositionally coherent. ALDEx2 measures the effect size and returns p-value as calculated by Wilcoxon test along with Benjamini-Hochberg (BH) adjusted p-value. Effect sizes are the ratio of the between-group differences to the maximum of within-group differences. For this analysis, we filtered-out rare and low abundant taxa further (recommended as to decrease sparsity in the dataset) by retaining only those taxa present in > 5% of the sample with mean abundance of > 0.01% in at least one of the two groups under comparison. We used a BH adjusted p-value cutoff of 0.1 for microbiome data and a BH adjusted p-value threshold of 0.1 and an effect size threshold of 0.80 for gene expression data.

### Host gene expression and pathway enrichment analysis

In the analysis of host gene expression data, we focused only on protein encoding genes, and we filtered out genes expressed in fewer than 25% of the samples for each disease type separately, retaining 9,925 unique genes for downstream analysis. As this dataset was also generated using NGS technologies, we applied a CLR transformation prior to conducting any downstream analysis.

To identify pathways found to be associated with a disease type, we implemented an enrichment analysis using Fisher’s exact test. We used the set of expressed genes input as the background genes and the set of genes associated with a disease type as the genes of interest. We used the KEGG and PID gene sets from the MsigDB canonical pathways collection.^35^ To avoid pathways that were too large to provide any specific biological insights or too small to provide adequate statistical power, we excluded from our analysis any pathways with more than 85 genes, fewer than 10 genes, or fewer than 5 genes that overlapped between the pathway and the genes of interest. The p-values obtained from Fisher’s exact test were adjusted for multiple testing using the Benjamini-Hochberg (FDR) approach.

### Host DNA methylation

As the epigenetic data was generated using two different arrays, all analysis conducted on this data type was repeated for each array separately. Prior to initial analysis, the raw beta values were normalized (to N(0, 1)) using Quantile Normalization. Principal Component analysis (PCA) was then conducted on this normalized DNA methylation data to identify any differences in methylation between groups under consideration. We also applied pairwise PERMANOVA test on the Euclidean distance matrix using 9,999 permutations to identify any community level differences between groups.

To identify CpG sites associated with disease-type, inflammation status and relapse status, either a linear mixed effect regression model (*lme4* package) or a linear regression model was used, depending on the underlying samples being used. If the underlying samples included paired samples from the same patient, a mixed effect model was used and the patient ID was included as a random effect. In both model types, condition, inflammation status, gender, age, methylation chip, sample position on methylation chip and biopsy location were included as fixed effects. Reported p-values were then adjusted for multiple testing using the Benjamini-Hochberg correction. To account for cell heterogeneity, this epigenetic association analysis was repeated for each set of significant epigenetic signals but this time incorporating the first 10 principal components (PCs) as covariates in the model. Only those CpG sites that were also significant (*p* < 0.05) based on the PC model were considered significantly associated with the outcome of interest (disease-type, inflammation status, relapse status).

Following this, we used the mCSEA package from Bioconductor to identify differentially methylated regions (DMRs) based on these significant CpG sites.^36^ In this analysis, we primarily focused on promoter regions and gene bodies. CpG sites were determined to be in a promoter region or gene body using the annotation R packages IlluminaHumanMethylation450kanno.ilmn12.hg19 and IlluminaHumanMethylationEPICanno.ilm10b2.hg19. Briefly, promoter regions were defined as those CpG sites whose *UCSC_RefGene_Group* column was either TSS1500, TSS200, 5’untranslate region [UTR] or 1stExon. CpG sites belonged to a gene body region if the *UCSC_RefGene_Group* was “Body”. Prior to identifying DMRs, CpG probes were first ranked using the *rankProbes()* function, with paired analysis conducted where necessary. Raw beta-values were input into this function and converted to M-values prior to calculating the linear models used to rank the CpG sites. In the cases where paired analysis was performed, the patient ID was supplied as the *pairColumn* parameter. Once CpG sites were ranked, the *mCSEATest()* function was used to identify differentially methylated regions (DMRs). Only those regions with an FDR-adjusted p-value <0.05 were considered differentially methylated.

### Integrating methylation and expression data

As both methylation and expression data were available for a subset of the cohort, we used the mCSEA package to integrate the two data types in order to identify significant associations between methylation changes in a DMR and expression alterations in a nearby gene. For this analysis, samples from methylation and expression datasets were matched by their sample ID, ensuring consistency with the patient ID, disease type, and inflammation status of the sample. Only those DMRs which were significant by *mCSEATest*, using the less stringent cutoff of a p-value less than 0.05, were considered in this analysis. The *mCSEAIntegrate* function was used to perform a correlation test between the mean DMR methylation and the expression of close genes. Only those regions with a correlation greater than 0.5 and an adjusted p-value less than 0.05 were recorded. By default, the package only reports negative correlations between promoter methylation and gene expression and positive correlations between gene body methylation and gene expression.

### Integration of host omics and gut microbiota data

We implemented a lasso penalized regression approach to identify specific associations between individual host features and gut microbial taxa as outlined in *Sambhawa Priya et al., 2022*. ^10^ Samples from different data types were again matched by their sample ID, as described previously. This was repeated for both host gene expression and host DNA methylation features. Given the large number of CpG sites included in the methylation arrays, we first merged the CpG sites into methylated regions using information from the *mCSEAdata* package. Similar to before, we considered only promoter and gene body regions and used the mean methylation value across the CpG sites associated with these regions to represent the methylation of that region. We implemented this analysis for each disease group (i.e., CD, UC and control) and their tissue biospecimen (i.e., inflamed or noninflamed) separately. For further analysis, we conducted correlation analysis on stability selected host genes-taxa associations. The Spearman correlation coefficient (rho) was used to depict the strength of association, while a Benjamini-Hochberg test was used to correct multiple testing problem. Pathway enrichment analysis for the host genes that were associated with specific gut microbes (q < 0.1) were carried out using Fisher’s exact test as outlined above.

### Succinotypes

The premise of succinotypes is that gut microbiomes typically have either *Phascolarctobacterium* or *Dialister* as their dominant succinate-consumer, and thus subjects can be classified as either P-type or D-type, respectively.^37^ Here, we classified the subjects into P and D types and tested for an association with disease.

To perform this classification, we first identified all RSVs that were classified on the genus level as either *Dialister*, *Phascolarctobacterium*, or *Phascolarctobacterium_A* using the assignTaxa function from the Dada2 package with the GTDBr95 database and an inclusive bootstrap cutoff of 0.2. This returned 7 RSVs, which we additionally aligned against the SSU references from GTDB with BLAST. All 7 RSVs had a perfect alignment to at least one of the references, confirming that the taxonomic classification was correct.

To assign succinotypes, we followed the same procedure as in Anthamatten et al.^37^ We then computed the read counts of *Dialister* (D) and *Phascolarctobacterium* (P) in each sample by summing the read counts of the respective RSVs and merging *Phascolarctobacterium* and *Phascolarctobacterium_A*. For each sample, we then computed the relative ratio of *Dialister* as *r*_D_ = *n*_D_/(*n*_D_+*n*_P_), where the *n* are the read counts of D and P, respectively. We assigned a clear D-type to a sample if *r*_D_ >0.9 and a clear P-type if *r*_D_ <0.1, implying 10× higher abundance of *Phascolarctobacterium vs. Dialister*, or *vice versa*. Samples with fewer than 10 combined D and P reads were not considered. If all samples from a subject had the same succinotype assignment, then this assignment was directly given to the subject. For subjects with discordant succinotype assignments between samples, we differentiated between those that had at least one clear assignment and otherwise mixed assignments (0.1 < r_D_ <0.9) and those that had fully discordant assignments. Those with clear and mixed assignments retained their clear assignment. One subject had fully discordant assignments, with and r_*D*_ = 1 in the fecal sample and r_*D*_ = 0 in all biopsies. For this sample, we assigned the succinotype of the biopsy.

We tested for associations between succinotypes and disease – both combined UC and CD or separate – and between whether patient had a relapse or remained in remission using Fisher’s exact tests. To test for an association between the number of relapses experienced by a subject and their succinotype, we initially used a non-parametric Mann-Whitney U-test. Following this, we implemented a zero-inflated Poisson regression approach to better understand the rate at which relapses occur. This model first splits the subjects into two groups: one in which the subjects are in long-term remission with a zero probability of relapses during the observation window (4 years) and a second in which relapses occur at a non-zero rate per year. A maximum likelihood estimation for different models types was performed to identify the models which best fit the data. We defined the (minus) log-likelihood function for the model by choosing a parametrization where we can estimate a joint theta (probability of long-term remission) for all groups or a separate theta value for each group. We always estimate a separate rate lambda (relapse rate) for each group. We compared models that grouped subjects based on succinotype, disease, succinotype and disease, and also CD/Dialister versus others. Once the best model was identified as CD/Dialister vs. others based on the Akaike Information Criterion (AIC), a log ratio test of this model versus one with a single lambda for all subjects was used to obtain a p-value for the differences in relapse rates between groups included in the model.

### ML analysis

ML analysis was conducted using the boosted decision tree algorithm, eXtreme Gradient Boosting (XGBoost).^38^ Six omics data types were considered in this analysis including three microbiome (16S gDNA, 16S cDNA and 16S G4 Phylochip) and three host-omics data types (host genotype, host RNA-Seq and host epigenome). We also considered the case where patient age was included as an additional feature.^39^ Models were trained using individual data types as well as multi-omics combinations with a total of 86 combinations assessed for each scenario (disease type and inflammation status).

Multi-omics datasets were combined using a concatenation approach and only samples for which we had full coverage across the data types were considered when training the model. Like previous analyses, samples from different data types were matched based on their sample ID and in the case of host genotype, were matched using patient ID. A full list of the available data types for each sample are provided in Supplementary Table S1. To reduce the dimensionality of the datasets, a number of feature reduction steps were considered. 16S gDNA, 16S cDNA and Host RNA-Seq data types underwent feature selection as described in earlier sections. We further reduced the dimensionality of both 16S sequencing datasets by agglomerating to genus level. For the host genotype, a subset of 264 SNPs which had been previously associated with IBD were considered.^5^ In addition to these feature reduction steps, only features with non-zero values in at least 10% of samples were considered and features with near-zero variance were removed. Similar to previous analyses, a CLR transformation was applied to NGS data types to account for the compositional nature of the data while, in this analysis, beta-values were used to represent the host epigenome dataset.

Given the limited sample size and the lack of an external validation set, a nested cross validation approach was implemented to train and assess the performance of our models (Supplementary Figure 13). The outer loop was a Leave-One-Out (LOO) Cross Validation (CV) and where more than one sample existed for a particular patient, all additional samples were excluded from the training data, to avoid introducing any bias into our pipeline. The held-out sample was used to assess the performance of the trained model and was not used for any other purpose. During the internal model development phase, on the training data an additional feature selection step was applied. A Wilcoxon rank-sum test was used to identify any potential associations between features and the outcome of interest. Those features found to have a significant difference (*p* < 0.05) between the relapse and remission groups were selected to be included in the training datasets. Model hyperparameters were selected using a randomized search based on a 5-fold CV. That is, we assessed the performance of 250 random combinations of hyperparameters using a 5-fold CV on the training data and those parameters with the highest mean area under the ROC curve (AUC) were chosen as the optimized hyperparameters. The following hyperparameters were tuned in each case *max_depth* (range: 5–80), *colsample_bytree* (range: 0.5–0.8), *subsample* (range: 0.5–0.8) and *alpha* (range: 0–150).

Once optimal hyperparameters were selected, 10 XGBoost models were trained on different subsets of the data by splitting the training set into 10-folds. Nine folds were used to train the model, and the remaining fold was used as model checkpoint and for early stopping. That is, if performance on this held-out fold did not improve after 20 rounds, training was stopped, and the model was saved. For the inference, the ensemble of 10 models was run and the average prediction from the 10 models was stored and was used to assess the performance on external held-out data. When splitting the training data into folds to create the ensemble of 10 models, predictions on the held-out *validation* fold were calculated for each model trained. These predictions represent the best achievable performance and were used to estimate the validation set performance of our ML approach. The AUC metric was primarily used to evaluate performance in each case (test and validation) as it is a threshold independent metric. Other threshold dependent metrics such as accuracy, sensitivity, specificity and F1-score were also calculated for the test set with the optimal threshold calculated using the geometric mean (G-mean) approach. The G-means is defined as the square root of the product of sensitivity (TPR) and specificity (TNR).

$$G-\mathrm{means}=\sqrt{\mathrm{Sensitivity}\times \mathrm{Specificity}}$$

The threshold for classification is selected such that the g-means value is maximized. As a result, this threshold represents the best trade-off between sensitivity and specificity, ensuring balance performance for both classes.

As part of the ML analysis, we also wanted to assess which features where playing a role in the trained models. This was done using two different techniques (i) feature importance (gain) from XGBoost models and (ii) Shapley additive explanation (SHAP) values.^40^ SHAP values show the contribution of each feature on a prediction of the model. In order to assess the overall impact of each omics type used in the model, we grouped the SHAP values by summing the values of all features in a particular omics dataset. Importance values for each feature, extracted from the trained XGBoost models, were averaged across each iteration of the CV and were normalized by the number of times it was found to have a non-zero value in an ensemble. This ML analysis was conducted using the following python packages: *xgboost, pandas, scikit-learn, numpy, shap* and *scipy*.

## Results

### Cohort demographics

The study cohort comprised 142 subjects with IBD, including patients with CD and UC, and 34 controls, as described previously.^5^ A total of 295 mucosal biopsies were collected from paired inflamed (i) and non-inflamed (ni) colonic sites from patients with IBD, and non-inflamed sites from control subjects (Table 1; Supplementary Table S1). The slightly higher number of samples compared with our previous study^5^ was due to the availability of new data types. We also chose to include a maximum of one inflamed and one non-inflamed biopsy sample per IBD subject (85% paired) and only one non-inflamed sample per control subject to avoid any biases that may arise from including multiple samples of the same type from the same patient. Stool samples were collected from a subset of the cohort (*n* = 39) for additional analysis (Table 1), whereof half of them were collected from patients on the same day as the colonoscopy (prior to bowel preparation) and the remaining within 3 years of biopsy collection. Additional patient information was gathered on disease related outcomes after the time of sampling (max 4 years), including future relapses, treatment with monoclonal antibodies, surgical intervention, and structural GI complications (Table 1). A patient was reported as having a relapse if there was a recurrence of symptoms and objective evidence of disease as assessed by endoscopy (sigmoidoscopy or colonoscopy) for patients with UC, and by endoscopy or CT scan for patients with CD.

**Table 1.** Subject characteristics, patient outcomes and data types of study cohort.

	Crohn’s Disease	Ulcerative Colitis	Controls	Total
Subjects	54	88	34	176
Biopsy Samples				
Inflamed	52	85	-	137
Non-Inflamed	50	76	32	158
Stool Samples	14	21	4	39
Age (Mean (SD))	41.6 (12.0)	47.9 (13.0)	55.7 (12.5)	47.5 (13.5)
Gender (M/F)	29/25	46/42	18/16	93/83
**Patient Outcomes 1–4 years after endoscopy (%)**				
Future Relapses (*n* = 140)	40.7%	43.0%	-	42.1%
Monoclonal antibody treatment (*n* = 134)	20.4%	11.3%	-	14.9%
Structural GI complications (*n* = 140)	22.2%	1.2%	-	9.3%
Surgical intervention (*n* = 142)	16.7%	2.3%	-	7.8%
**Number of patients on Medication at time of sampling**				
Biologics	6	10	-	16
Corticosteroids	7	11	-	18
Mercaptopurine	13	6	-	19
Mesalazine	5	36	-	41
Nexium	2	2	5	9
**Data types: Biopsy**				
16S gDNA	85	131	30	246
Inflamed (i)	42	66	-	108
Non-inflamed (ni)	43	65	30	138
16S cDNA	83	133	25	241
Inflamed (i)	45	73	-	118
Non-inflamed (ni)	38	60	25	123
Host RNA-Seq	87	132	26	245
Inflamed (i)	44	71	-	115
Non-inflamed (ni)	43	61	26	130
Host DNA Methylation (Epigenome)	74	76	23	173
Inflamed (i)	37	30	-	67
Non-inflamed (ni)	37	46	23	106

### Microbiota composition of IBD subtypes is different to controls

We examined the mucosal microbiota using amplicon sequencing of both 16S rRNA genes (gDNA or standing microbiota; Table 1) and transcripts (cDNA or active microbiota; Table 1). The latter amplicon was added because gDNA cannot differentiate between dead or alive cells^7^ and also because it captures the metabolically active microbial community for a more functional view. 16S gDNA data from stool samples, collected as described previously, was also available for a sub-cohort (14 CD, 21 UC, 4 controls) allowing us to compare microbial composition between sample types for available patients (Table 1). As these samples were not collected in an RNA preservative, 16S cDNA data was not available.

Microbiota analysis was carried out on a total of 11.4 million error-corrected, non-chimeric ribosomal sequence variant (RSV) reads with a mean count of 21,686 ± 8,667 SD using updated methods from previous analysis^5^ (Supplementary Figure S1). In total 12,006 unique RSVs were identified across all 16S rRNA amplicon data types, whereafter filtering for those present in at least 5% of samples in at least one dataset (gDNA biopsy, cDNA biopsy, gDNA stool) resulted in a unique set of 520 RSVs. Any samples with less than 5,000 reads were also removed from further analysis.

**Figure 1.**
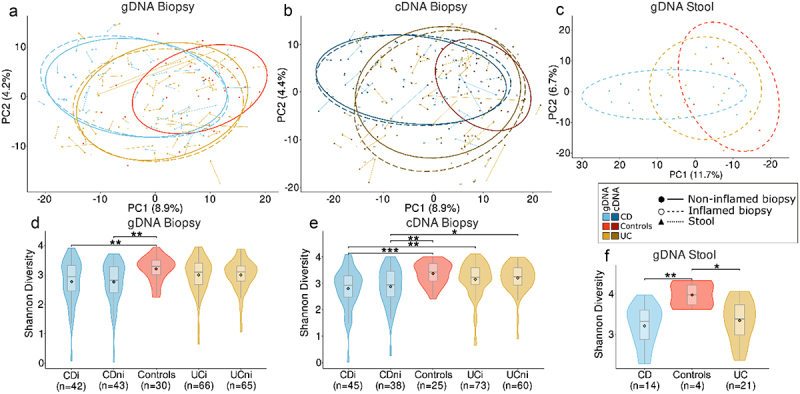
Microbiota composition and diversity of Crohn’s disease (CD), ulcerative colitis (UC) and control subjects. Principal component analysis (PCA) based on Aitchison distances of all RSVs present in > 5% of samples in at least one dataset under consideration for (a) gDNA biopsy, (b) cDNA biopsy and (c) gDNA stool datasets, respectively. Samples are grouped by disease type and inflammation status. Points connected by lines highlight samples from the same patient. d-f) Comparison of alpha diversity using the Shannon diversity index for each 16S rRNA dataset. Diversity is compared for each disease type and inflammation status. *p < 0.05, **p < 0.01, ***p < 0.001.

Unlike the original study,^5^ beta diversity analysis was performed based on Aitchison distances to better account for the compositional nature of the data. We observed a significant (PERMANOVA, *p* < 0.05) disease-associated shift in microbiome composition along the first two principal components (PCs) for both mucosal 16S rRNA datasets, with CD samples showing the most significant shift from control samples (Figure 1a-b; Supplementary Table S2). The microbiota composition of available stool samples also showed a significant disease-associated shift, with differences observed between CD and controls, and between CD and UC subjects (Figure 1c). Again, CD samples showed the most significant shift away from controls. For a subset of patients with CD (*n* = 29), we had information on ileal involvement in their disease. Beta diversity analysis showed a significant PERMANOVA difference in the standing microbiota of CDi samples from those patients with ileal involvement and those without (Supplementary Figure S2a). This was not observed when examining the active microbiota and no significant difference was observed between these groups when analyzing non-inflamed samples for either data type (Supplementary Figure S2a-b).

In line with previous findings,^5^ CD mucosa exhibited a lower microbial diversity than controls in 16S gDNA data, while inflammatory status of samples did not significantly affect diversity levels (Figure 1d; CDi vs controls *p* = 0.02; CDni vs controls *p* = 0.01). These differences were also evident for active microbiota (Figure 1e; CDi vs controls *p* = 0.001; CDni vs controls *p* = 0.004), even though CD samples also demonstrated significantly lower diversity than UC samples (UCi vs CDi *p* = 0.005; UCni vs CDi *p* = 0.003). In contrast to the previous study,^5^ there was no significant difference in alpha diversity between UC and controls for either amplicon data type (Supplementary Table S2). In stool samples, both CD and UC patients displayed a significantly lower alpha diversity compared to controls (Figure 1f: CD vs controls *p* = 0.008; UC vs controls *p* = 0.049). However, no difference in diversity was found between UC and CD stool samples, or for the subset of patients with known ileal involvement (Supplementary Figure S2c-d).

**Figure 2.**
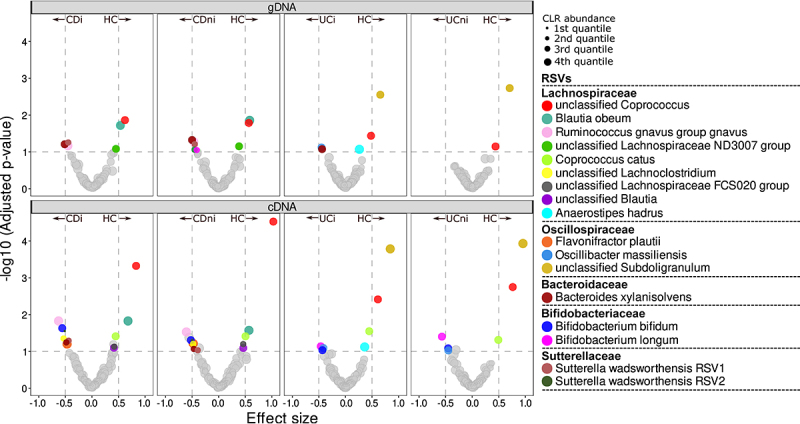
Volcano plots of differential abundance analysis comparing RSV abundances of CD and UC subjects to controls. Analysis was repeated for each inflammation status and 16S rRNA data type. Points above the horizontal line represent those taxa with an adjusted p-value (q) < 0.1 and those outside the vertical lines have an effect size >±0.5. Species abundance is denoted by point size where 4^th^ quantile denotes that the species is in the least abundant category.

Differential abundance analysis of RSVs from mucosal samples was performed using compositional-aware ALDEx2 to compare IBD and controls (Figure 2). While it was possible to detect several differentially abundant taxa, these findings were not always consistent across amplicon datasets. For all comparisons of disease type and inflammation status, only an unclassified *Coprococcus* RSV (previously identified as unclassified *Lachnospiraceae)* ^5^ was consistently less abundant in both IBD subtypes compared to controls across both 16S data types. *Blautia obeum* and unclassified *Subdoligranulum* RSV were also found to be less abundant in CD and UC, respectively, when examining both active and standing abundances (Figure 2 & Supplementary Figure S3). In contrast to previous analyses,^5^ we did not find a significant difference in *Anaerostipes hadrus* abundance when comparing CD to controls. It was, however, less abundant in UC compared to controls in terms of statistical significance, but the effect size was below the chosen threshold (Figure 2). No significant differences were observed between inflamed and non-inflamed mucosa.

**Figure 3.**
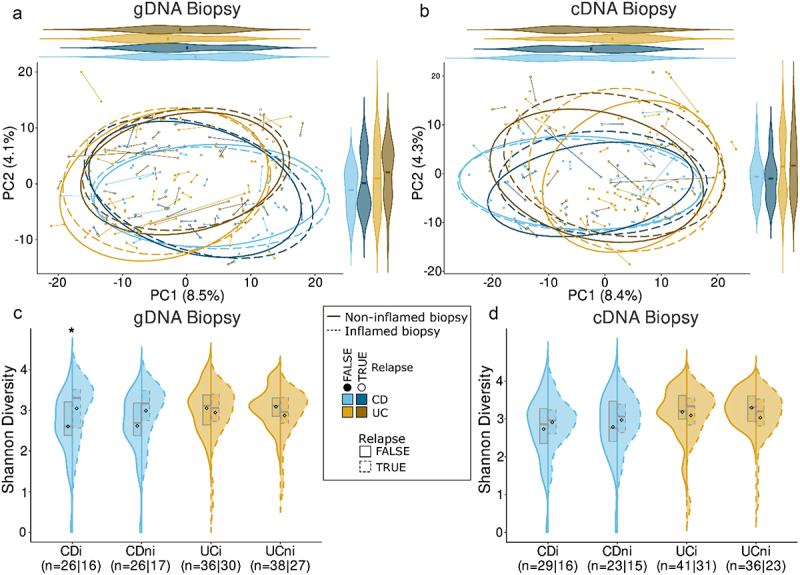
Comparison of microbiota composition and diversity between patients with IBD who experienced a relapse of disease and those who remained in remission within 4 years of sampling. (a-b) Principal component analysis (PCA) based on Aitchison distances grouped by relapse status, inflammation status and disease type with analysis repeated for 16S gDNA and cDNA datasets, respectively. (c-d) Comparison of Shannon alpha diversity between relapse and remission groups for gDNA and cDNA datasets respectively. *p < 0.05.

To add an outcome-predictive component, we repeated the above analysis on patients that had at least one relapse within four years of endoscopy sampling and those that remained in remission. Beta diversity was not significantly different between relapse and remission for either 16S amplicon datasets (Figure 3a-b; Supplementary Table S3). Somewhat counterintuitively, relapsing patients with CD had a higher alpha diversity in their inflamed samples compared to those in remission (*p* < 0.05; Figure 3c; Supplementary Table S3). This was however only observed when considering 16S gDNA abundances and not corroborated in active microbe abundances (Figure 3d). Given the limited number of CD subjects with information on ileal involvement and relapse status, we could not compare these groups. Similarly, we found no bacterial taxa to be differentially abundant in either relapse or remission after adjusting for multiple testing (see Supplementary Figure S4 & S5 for significant RSVs before adjustment).

### Integrating host omics data with the gut microbiome

**Figure 4.**
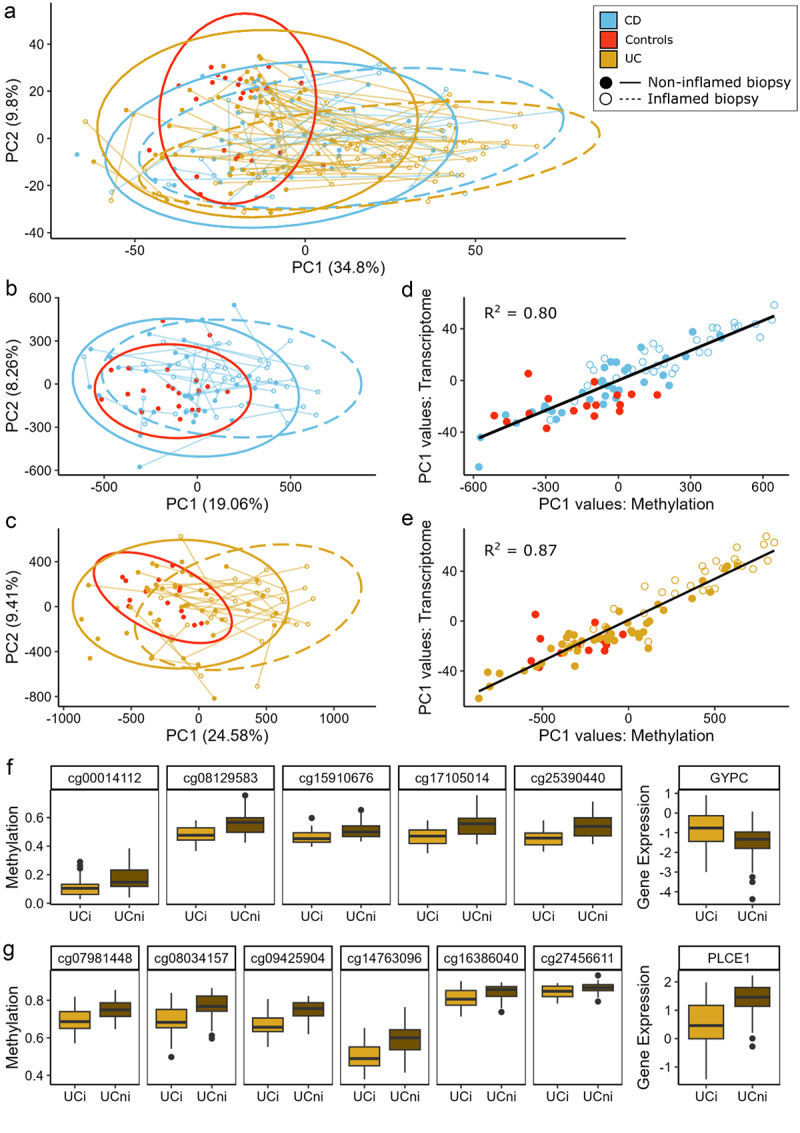
Host gene expression and DNA methylation in Crohn’s disease (CD), ulcerative colitis (UC) and control subjects. (a) PCA plot based on Aitchison distances of Host RNA-Seq data grouped by disease type and inflammation status of samples. Points connected by lines highlight those samples from the same patient. (b-c) PCA plots of Host epigenetic data grouped by disease type and inflammation status for those samples generated using the 450K and EPIC methylation arrays, respectively. (d-e) Plot of PC1 values comparing Host methylation and Host transcriptome for each methylation array. (f) Boxplots of methylation of CpG sites associated with the promoter region of the *GYPC* gene and its corresponding gene expression. (g) Boxplots of methylation of CpG sites associated with the gene body of *PLCE1* and its corresponding gene expression.

In addition to microbiome data, host omics datasets were also generated from the same mucosal biopsies,^5^ including host transcriptome (245 biopsies from 147 patients; Table 1) and host epigenome data (173 biopsies from 106 subjects; Table 1). As outlined in methods, host epigenome samples were generated on two different Illumina arrays and due to this batch effect, we conducted all epigenetic analysis separately for each array. We observed significant disease-associated shifts in gene expression and methylation between patients with IBD and controls with CDi samples being the furthest away from controls (Figure 4a-c; PERMANOVA *p* < 0.05; Supplementary Table 4 &5). Significant inflammation-associated changes in host omics were also identified within both CD and UC samples (Figure 4a-c; *p* < 0.05; Supplementary Table 4 &5) and this was corroborated by a strong epigenome-transcriptome correlation between the inflammation-associated PC1 values (Figure 4d-e; 450K array R^2^ = 0.8; EPIC array R^2^ = 0.87).

Differential expression analysis further highlighted this inflammation-associated change in gene expression, with 704 and 1,134 differentially expressed genes (DEGs) identified in CDi and UCi samples, respectively, compared to controls (q < 0.05; effect size>±0.8; Supplementary Table S6 & 7). These DEGs corresponded to 66 enriched pathways of which 29 were common to both IBD subtypes and included pathways involved in fatty acid, amino acid and carbohydrate metabolism, along with integrin and interleukin pathways (Supplementary Figure S6). The central regulator gene ETS2, recently reported as causative of macrophage inflammation in IBD,^41^ was also among those DEGs upregulated in IBD. There were, however, no differences in expression across patient groups with the corresponding risk SNP (*rs2836882*).

Examination of individual CpG sites using mixed-effect models also highlighted the difference in methylation by inflammation status within IBD subtypes. We identified 14,601 and 35,322 CpGs that were significantly associated with inflammation in CD and UC samples, respectively. These inflammation-associated CpGs corresponded to three differentially methylated promoter regions (*FLJ44606, UTS2D, HTR2A*, q < 0.05) and one differentially methylated gene body (*HLA-DPB1*) in CD and 19 promoter regions and 11 genes bodies in UC (Supplementary Table 8). Using the mCSEA package^36^ and a less stringent cutoff for differentially methylated regions (DMRs; *p* < 0.05), it was possible to examine the connection between methylated regions and the corresponding gene expression. In UCi samples, we found 9 promoter regions and 10 gene bodies whose methylation was significantly correlated with the expression of the corresponding gene (Supplementary Table 9). For example, a strong negative correlation was observed between the methylation of CpG sites in the promoter region of the *GYPC* gene and its corresponding expression (Figure 4f; ρ=-0.74) while a strong positive correlation was found between the expression and methylation of the PLCE1 gene (Figure 4g; *p* = 0.8). In CDi samples, a moderately strong positive correlation was reported between the methylation and expression of genes *AGAP1* (ρ = 0.66; Supplementary Figure 7) and *PTPRN2* (ρ = 0.65; Supplementary Figure 8).

As matched 16S amplicon data with host RNA-Seq data (215 cDNA and 209 gDNA) and host epigenome data (160 gDNA and 141 cDNA) was available for a subset of samples, we implemented a penalized regression approach to elucidate individual host-microbe associations.^10^ Given the large number of CpG sites produced by Illumina arrays and resulting multiple testing issues, we merged these sites into methylated promoter and gene body regions. Enrichment analysis of host gene expression identified relationships between 54 genes and six bacterial taxa across eight pathways (Table 2; Fisher’s exact test; q < 0.1). Of all gene-microbe associations identified, only one pathway was significantly enriched based on associations with both the standing and active microbiota of the same taxa. In UC patients, we found the expression of genes from the Integrin beta-1 pathway to be positively correlated with both the standing and active abundance of the *Parasutterella* genus (Supplementary Figure 9).

**Table 2.** Pathways enriched for significant gene-microbe associations.

Pathway	DB	Taxa Name (Level)	q	# of Genes	Genes Names
**CD Inflamed Samples (gDNA)**					
Cardiac Muscle Contraction	KEGG	*Ruminococcus torques group torques* (RSV)	****	14	*COX4I1, COX5B, COX6A1, COX6B1, COX7A2, COX7A2L, COX7B, COX7C, COX8A, UQCR10, UQCR11, UQCRB, UQCRH, UQCRQ*
Proteasome	KEGG	*Ruminococcus torques group torques* (RSV)	*	9	*PSMA7, PSMB1, PSMB3, PSMB7, PSMC5, PSMD13, PSMD4, PSMD8, SEM1*
**CD Inflamed Samples (cDNA)**					
RNA Degradation	KEGG	*Bifidobacterium longum* (Species)	***	6	*CNOT6L, CNOT7, DCP2, DDX6, EXOSC4, PAPOLA*
P53 Signalling Pathway	KEGG	*Bifidobacterium longum* (Species)	**	5	*CCND3, MDM4, PTEN, RRM2B, SESN3*
Cardiac Muscle Contraction	KEGG	*Unclassified Lachnoclostridium* (RSV)	***	5	*COX5A, COX5B, COX6B1, COX7A2, COX8A*
**UC Inflamed Samples (gDNA)**					
Integrin 1 Pathway	PID	*Parasutterella* (Genus)	****	8	*COL6A3, COL7A1, ITGA5, LAMA5, LAMC1, LAMC2, TGFBI, TNC*
Aurora B Pathway	PID	*Parabacteroides distasonis* (RSV)	****	6	*BUB1, KIF20A, KIF23, KIF2C, NCAPD2, NCAPH*
**UC Inflamed Samples (cDNA)**					
Inositol Phosphate Metabolism	KEGG	*Veillonellaceae* (Family)	**	5	*INPP5E, INPPL1, PIK3CB, PIP5K1C, PLCG1*
Phosphatidylinositol Signalling System	KEGG	*Veillonellaceae* (Family)	**	5	*INPP5E, INPPL1, PIK3CB, PIP5K1C, PLCG1*
Integrin 1 Pathway	PID	*Parasutterella* (Genus)	****	5	*COL7A1, ITGA5, LAMA5, LAMC2, TNC*

****q < 0.0001; ***q < 0.001; **q < 0.01; *q < 0.1.

We identified links between the methylation of 64 promoter regions and two microbial taxa across 27 pathways in CDi samples (Fisher’s exact test q < 0.1; Supplementary Figure 10). A total of 21 pathways were enriched based on promoter regions associated with the standing abundance of the *Lachnospiraceae UCG-004* genus, and a further six pathways were associated with the standing abundance of a *Lachnospiraceae CAG-56* RSV. None of these associations were significant when considering the active abundances of these taxa and no enriched pathways were identified based on promoter-microbe associations in UC samples. No pathways were enriched based on associations between methylated gene bodies and microbes for either IBD subtype.

As with the microbiome analysis, we compared relapse and remission groups within both IBD subtypes and omics types but found no significant differences in overall gene expression or methylation between these groups in both CD and UC (Supplementary Table 4 & 5). No DEGs met our significant threshold once we adjusted for multiple testing. When considering methylation data for inflamed and non-inflamed samples together, 3,584 CpGs were significantly associated with relapse status in CD and 590 CpGs in UC subjects (q < 0.05; Supplementary Table 10). However, once samples were split by inflammation status, no CpGs remained significantly associated with relapse, after adjusting for multiple testing.

### Succinotypes of patients with IBD are associated with number of future relapses

Recent work has showed that individuals can be partitioned based on their gastrointestinal succinotype, i.e. the taxonomic identity of their dominant succinate-consuming bacterium, either *Dialister* (D) or *Phascolarctobacterium* (P).^37^ Succinate can act as a pro-inflammatory signaling molecule, which when produced above a certain threshold in the colon can contribute to starting and/or maintaining an inflammatory signaling cascade.^42,43^ In patients with IBD, the slower succinate-consuming D-succinotypes have been reported as overrepresented compared to healthy controls, suggesting a potential contribution of succinate removal to pathophysiology.^37^ We therefore wanted to assess the distribution of these dominant succinotypes in our patients with IBD and how they may relate to future relapse.

**Figure 5.**
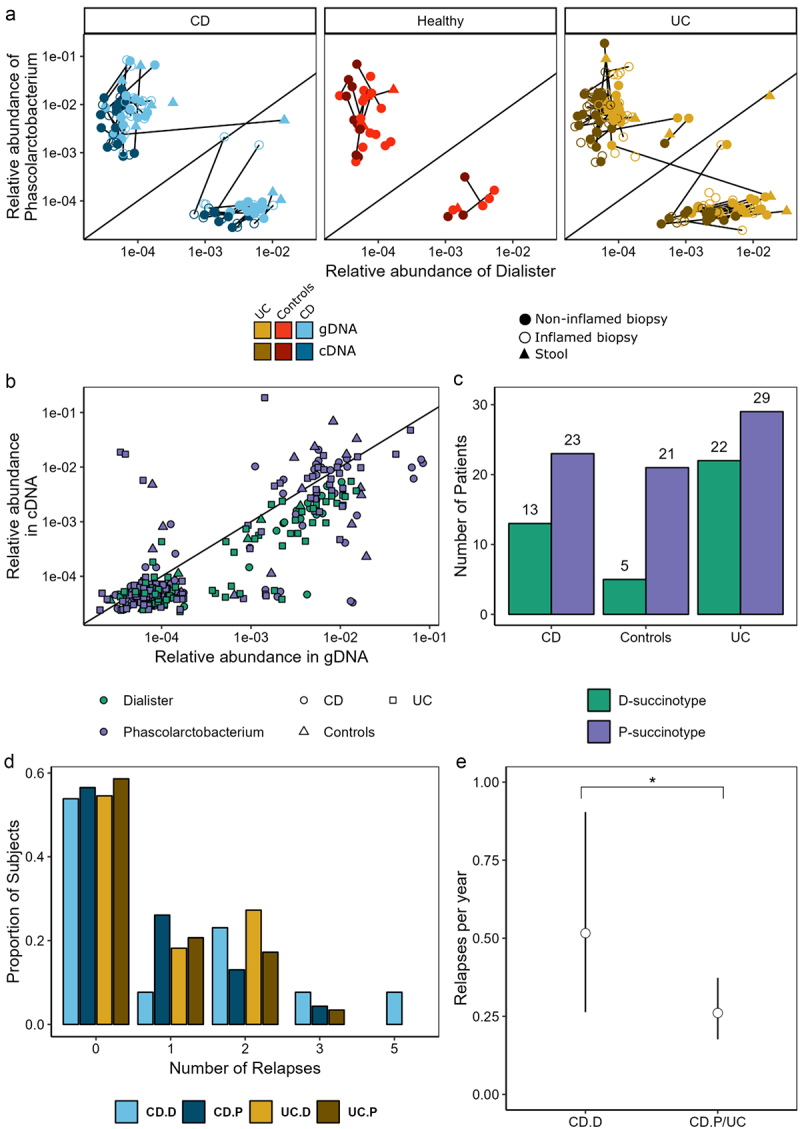
Succinotypes can be defined in CD, UC and control subjects. (a) Relative abundance of *Dialister* vs *Phascolarctobacterium* within samples for both standing and active datasets. (b) Comparison of the relative abundance of genera in gDNA and cDNA. (c) Bar plot of succinotypes grouped by disease-type. (d) Number of relapses by disease and succinotype. (e) Relapses per year with 95% confidence intervals for CD.D vs all other groups estimated using zero-inflated poisson model. *p < 0.1.

Here, we identified 7 RSVs in both 16S rRNA datasets that taxonomically classified as either P or D and verified that these RSVs mapped perfectly to known representatives of the respective genera (see Methods). The grouped relative abundances for both standing and active abundances P and D were indeed bimodally distributed with strong mutually exclusivity between the two (Figure 5a-b; rhoD = 0.892; rhoP = 0.812), consistent with the concept of succinotypes. Following Anthamatten et al. (2024),^37^ we subsequently assigned succinotypes to samples based on the relative proportion of Dialister, r_D_, defined as D counts divided by the sum of D and P counts. A sample was assigned either D- or P-succinotype if r_D_ >0.9 or r_D_ <0.1, respectively, and there were at least 10 reads assigned to D and P. A substantial number of samples did not have any reads assigned to either D or P, though this proportion was notably higher for the biopsies (42%; 207/487) than for the stool samples (21%; 8/39) (Fisher’s exact test, *p* = 0.007). We checked for the consistency in succinotype assignment for an individual by comparing across all respective samples (gDNA/cDNA, inflamed/non-inflamed). Only one single individual had discordant succinotype assignments across samples, where the fecal sample was a D-succinotype and the three biopsy samples were P-types. In nine other individuals, samples had non-zero counts of both D and P, but the remaining 103 individuals had consistent succinotype assignments across all samples (Figure 5c). We thus concluded that succinotypes were also well-defined for the samples used here and were able to assign succinotypes for 113 of the 175 subjects.

The distribution of succinotypes across disease types was marginally significantly different between IBD (CD+UC) and controls (Figure 5c; Fisher’s exact test, *p* = 0.062). Once split into CD and UC, we found no significant difference in succinotypes between CD and controls (*p* = 0.170), but did notice a significant difference in distribution between UC and controls (*p* = 0.046). There was no difference in the proportion of subjects with and without future relapses between succinotypes, both in UC (*p* = 0.784) and CD (*p* = 1). We did, however, observe a trend for a higher number of relapses in CD patients with the D compared to the P succinotype (patients with at least one relapse, Mann-Whitney U test, *p* = 0.080), but not in UC (*p* = 0.82). To more carefully evaluate this trend, we fitted a zero-inflated Poisson model to the number of relapses (Figure 5d). The zero-inflation accounts for a certain probability of relapse during the observation window (4 years), and the Poisson distribution models that when patients do have a relapse, these relapses occur with a certain rate. We did not find any significant succinotype differences in terms of the probability of having a relapse. However, we did observe that patients with CD who had the D-succinotype had a significantly higher relapse rate (mean 0.51 relapses/year; *p* = 0.059), while P-types and UC patients had lower relapse rates (mean 0.26 relapses/year; Figure 5e). This suggests that the D-succinotype is potentially associated with a higher frequency of relapses.

### Prediction of relapse using machine learning

As it proved difficult to distinguish between future relapse and remission using traditional statistical analysis of single omics datasets (with the exception of associating succinotypes with number of relapses), we employed the ML method Extreme Gradient Boosting (XGBoost) to see if we could better predict these two groups using combinations of the omics datasets available. This powerful ML algorithm has already shown promising results in different areas of omics research due to its ability to handle different data types and missing data as well as its easy interpretation.^5,11,44–46^ In addition to the biopsy datasets used above, we included two datasets generated from the same samples that did not previously have sufficient power for single omics analysis.^5^ This resulted in three host omics data types (transcriptome, genotype, epigenome) and three microbiome data types (16S gDNA and cDNA genera and 16S gDNA G4 Phylochip (eOTUs)). The XGBoost models were trained on data from inflamed, non-inflamed and paired samples from UC and CD subjects. In each case, the analysis was performed as part of a cross-validation (CV) performance assessment routine, where an ensemble of 10 XGBoost models was used as a predictor (see Methods).

**Figure 6.**
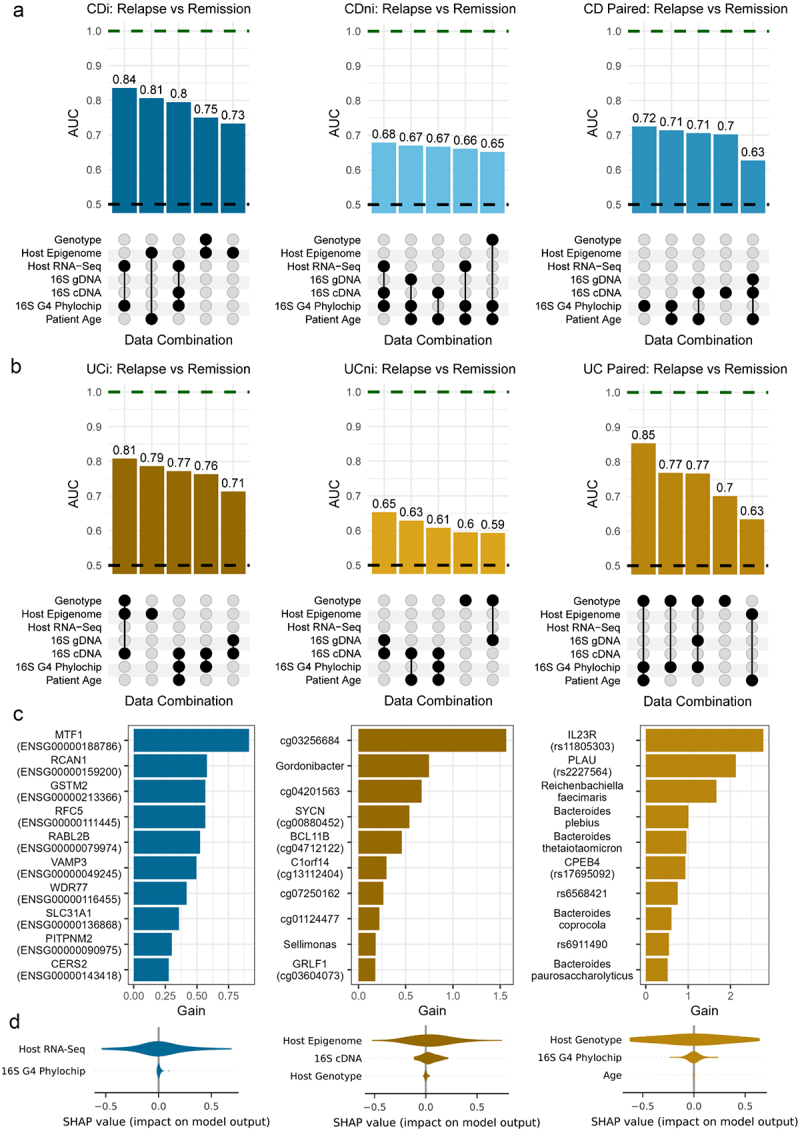
Outline of XGBoost model performance to predict relapse in (a) Crohn’s disease (CD) and (b) ulcerative colitis (UC) subjects. UpSet plots outline AUCs and the combination of features used to achieve model performance. Top row shows top 5 models when predicting future relapse in patients with CD, where models were trained on inflamed, non-inflamed and paired data, respectively (left-right). Second row shows top 5 models when predicting future relapse in patients with UC. Green dashed line indicates perfect performance. Black dashed line is equivalent to a random model. (c) Top 10 important features based on gain importance metric from XGBoost. Features presented are those from the highest performing model when models were trained on CDi, UCi and UC paired samples, respectively (left-right). (d) SHAP values extracted from same models as c) but values were grouped (summed) by those omics types used to train the model.

Models trained on inflamed CD samples had, in general, better performance than those trained on CD non-inflamed or paired samples (Figure 6a; Supplementary Table 11). The highest Area Under the ROC Curve (AUC) was achieved when predicting relapse using both host RNA-Seq and 16S gDNA G4 Phylochip features (AUC = 0.84). We also observed promising performance from a model trained on host epigenome features combined with patient age (AUC = 0.81). In both cases, the datasets were generated from the inflamed mucosal samples of patients with CD. For models trained on either non-inflamed or paired data, the highest AUCs achieved were 0.68 (Host RNA-Seq +16S cDNA + 16S G4 Phylochip) and 0.72 (16S G4 Phylochip), respectively. Additional performance metrics for these models, including accuracy, sensitivity, specificity and F1-score, are provided in Supplementary table 11.

Models also performed well in predicting relapse in UC when using either inflamed or paired data (Figure 6b; Supplementary Table 12). The highest AUC for UC was achieved by combining features from host genotype and 16S gDNA G4 Phylochip datasets in conjunction with age when trained on paired inflamed and non-inflamed samples (AUC = 0.85). A model trained using multi-omics data from inflamed mucosal samples also showed high performance, achieving an AUC of 0.81 (host genotype, host epigenome and 16S cDNA datasets). When considering UC and CD patients together (IBD), we saw lower performing models than when each subtype was considered separately (Supplementary Figure 11; Supplementary Table 13). Given the complexity of multi-omics datasets and lack of an external validation dataset, we assessed the generalizability and stability of our models by comparing the performance of our model on the validation set (inner loop of CV) and the test set (outer loop of CV), see Supplementary Information 1.

To add an interpretative component, we elucidated which features were having the biggest role in the higher performing models. We therefore examined both feature importance values extracted from XGBoost models and SHapley Additive exPlanation (SHAP) values, which highlight the contribution of each feature on a prediction of the model (Figure 6c-d). Based on these results, the expression of *MTF1* and *RCAN1* genes were the most important features when predicting relapse in CD subjects based on inflamed sample data. For UC, the CpG site cg03256584 and the SNP rs11805303 of the pro-inflammatory gene *IL23R* were the most important when models were trained on inflamed and paired data, respectively. Also among the top 10 features for UC subjects were microbial features such as the active genera *Gordonibacter* and *Sellimonas* (inflamed model) and species such as *Bacteroides plebeius* and *Bacteroides thetaiotaomicron* (paired model), which have all been previously associated with IBD.^47–51^ In addition to considering feature importance, we calculated SHAP values for each model which we grouped by omics types. Here, grouped SHAP values indicated that host omics data had the largest impact on predictions for each of the top models outlined previously (Figure 6d).

## Discussion

The potential causes of immune-mediated diseases such as CD and UC are widely considered multi-factorial implicating both human genetics and the gut microbiota, thus requiring corresponding omics types to investigate relevant host-microbe interactions.^1,52^ In this study, we examined a comprehensive multi-omics dataset from mucosal biopsies to get further insight into IBD subtypes and the future disease states of these patients. We considered both single omics and integrative approaches to highlight differences in the disease types and their relapse states.

Our analysis showed differences in microbial composition between IBD subtypes and controls, which was consistent with existing literature.^5,7,53^ This was further expanded upon to include, not only the standing microbiota abundances, but also the metabolically active microbes. We observed similar trends for both 16S rRNA data types in terms of alpha and beta diversity, with CD patients having the greatest difference in composition compared to controls. These trends were more pronounced in the active microbiota, emphasizing their potential importance. Taxa such as a *Coprococcus* species, *Blautia obeum*, a *Subdoligranulum species* were consistently differentially abundant in disease relative to controls across both data types, and all of these taxa have been previously associated with IBD.^5,54–56^

Simultaneously considering both 16S rRNA data types has only been done once previously in the context of IBD.^7^ The authors in that study (*n* = 89) reported a significant reduction in the ‘active’ alpha diversity of CD subjects compared to healthy, consistent with our findings. In another IBD multi-omics study, the authors compared 78 paired fecal metagenomes and metatranscriptomes,^57^ highlighting more pronounced results in their active (metatranscriptomics) data, broadly consistent with our findings.

Our analysis of host omics data highlighted differences between IBD and control subjects with inflamed IBD samples showing the largest change in both expression and methylation. Enrichment analysis of DEGs showed many significant pathways many of which were common to both IBD disease-types, including fatty acid, amino acid, and carbohydrate metabolism, along with integrin and interleukin pathways (IL-23 and IL-12), all of which have well established associations with IBD.^58–63^ Our examination of methylation regions between inflamed and non-inflamed samples highlighted several DMRs significantly correlated with the corresponding gene expression. Methylation of the promoter region of the *GYPC* gene had a strong negative correlation with its gene expression in UC patients. This gene has previously been associated with response to corticosteroid therapy in pediatric UC patients.^64^ Similarly, a strong positive correlation was found between the methylation and expression of *PLCE1* in UC, which was previously linked to this disease.^65^ A subgroup of patients with UC was defined by the genes *SLC4A4, EPB41L4B* and *PLCE1* with patients in this subgroup reported to have milder clinical condition, but more likely to progress to colorectal cancer.^65^ We also observed moderate correlations between methylation and gene expression for genes such as *AGAP1* and *PTPRN2* in CDi samples. To our knowledge, no direct associations between *AGAP1* and CD have previously been made, however, several studies have observed links between *PTPRN2* and CD.^66,67^ For example, studies have found *PTPRN2*, a gene which encodes the protein tyrosine phosphatase, to be differentially methylated in both adipose stem cells^66^ and peripheral blood cells of patients with CD.^67^

As both microbiome and host omics data was available for most of our cohort, we supplemented the single omics analysis by examining the interplay between individual host features and different taxonomic levels of gut microbes. We were able to identify associations between several microbes and various metabolic pathways for both CDi and UCi samples. Interestingly, the integrin beta-1 pathway was significantly enriched based on genes associated with the abundance of the *Parasutterella* genus in UC patients for both the standing and active abundances. Genes from the integrin beta-1 pathway had previously been associated with taxa such as *Dialister, Phascolarctobacterium*, and *Intestinibacter* in subjects with IBD.^10^ *Dialister, Phascolarctobacterium* and *Parasutterella* all play a role in controlling the level of succinate present in the gut with the two former being known succinate consumers and the latter a succinate producer.^68,69^ Interestingly, when we examined the distribution of the dominant succinate consuming bacteria in our cohort, UC patients had a different distribution of succinotypes compared to controls while no difference was observed between CD and control subjects.

A common finding across our single omics analyses was that while we could identify differences between disease types, it proved more difficult to distinguish future relapse and remission. Only CDi samples (gDNA only) of those subjects that reported a relapse had a significantly different alpha diversity compared to CDi samples of those that remained in remission. No other differences in terms of alpha/beta diversity, taxa abundances or host gene expression were found when comparing the two outcome groups. Similarly, other investigators did not find significant microbial diversity differences between relapse and remission (stool metagenomics) in UC or CD subjects.^55^

While most of our single omics analyses fell short of significantly distinguishing between future relapse and remission, we did observe that CD patients with a D-succinotype had a significantly higher number of relapses per year. Succinate has been implicated in IBD pathology, for example by perpetuating a pro-inflammatory state in macrophages^70^ or contributing to the formation of fistulas.^71^ However, having a D-succinotype alone is not sufficient to cause disease as both D and P types are evenly distributed in healthy individuals.^37^ *Dialister* consumes succinate more slowly compared to *Phascolarctobacterium*, leading to higher intestinal succinate concentrations in D-succinotype individuals compared to P ones. Thus, it is conceivable that other factors contribute to the onset of disease activity, which in turn is exacerbated by higher intestinal succinate concentrations resulting in flares. So not only is the slower succinate-removing D-succinotype more common in IBD,^37^ as is the increased abundance of *Dialister invisus* in general in IBD compared to non-IBD patients,^8^ but our findings also indicate predictive potential for future relapse frequency.

Encouragingly, it was possible to achieve good performance in predicting relapse by applying a ML approach to multiple omics datasets, in particular from inflamed samples. The highest performance was achieved when combining both host and microbial data, highlighting the host-microbial importance of any predictive profiles. While previous studies have attempted to predict relapse in IBD, the definition of relapse often differs and very few studies include more than one omics types in their analysis. Most studies applying ML to multi-omics data did so in order to classify disease.^5,11,72,73^ Sarrabayrouse and colleagues combined baseline microbiota and fungal loads from qPCR measurements of stool samples, inflammatory markers and flare history to predict relapse one year later for both CD and UC.^13^ It is however not surprising that clinical meta-data like previous flare history can significantly improve prediction of future relapse. Protein and metabolomics biomarkers in serum in another IBD study were associated with relapse within two years by using logistic regression.^12^ Based on stool microbiota composition alone, an AUC of 0.67 was obtained when predicting onset of CD within 5 years in healthy first-degree relatives of patients with CD.^6^ None of these studies based their predictive models on integrated host-microbial molecular data from intestinal mucosa. However, the predictive performances achieved in our study appear comparable to those reported in existing literature with the performance of many models matching or surpassing those already published.

Although, we are seeing promising relapse-predicting results in terms of our succinotype and ML analyses, there are some limitations to our study. Firstly, our cohort consisted of only adult patients with IBD who were not newly diagnosed and therefore not treatment naive. As a result, our findings may not fully account for potential biases introduced by long-term illness and exposure to various treatments. In future studies it may be beneficial to examine treatment-naïve patients to assess if baseline features may be predictive of future relapse or else collect extensive meta data on patient clinical features such as past and present medication. Secondly, our dataset consisted of single time-point data which was used to predict future relapse. A longitudinal approach may be more informative as it would be possible to follow the trends in each omics dataset across multiple time points and disease states.

While we had a large cohort of patients, not all data types were available for all samples and in many parts of our analysis only those samples with full coverage were considered. Consequently, our ML analysis was conducted using a nested cross validation approach and could not be validated as no suitable external dataset was available. While finding an equally comprehensive multi-omics study with future outcome data will be challenging, our findings should ideally be externally validated on new subjects when a prospectively recruited suitable validation cohort becomes available. Additionally, while we recognize that generating this type of multi-omics dataset may not be feasible for some researchers or in clinical settings due to cost and sample constraints, we hope our analysis will be helpful in guiding future studies by highlighting potentially more informative data types. Once validated, these models could then progress the development of prognostic tools in a clinical setting.

In conclusion, in contrast to a single omics approach, multi-omics analysis incorporating both host and microbiome data was predictive of clinical relapses with IBD. Future validation studies could next be designed with the best performing omics data in mind, which could eventually progress the development of prognostic tools.

## Supplementary Material

Supplemental Material

## Data Availability

Sequence data and array data generated as part of the original study are available at NCBI BioProject (https://www.ncbi.nlm.nih.gov/bioproject/.) PRJNA398187 and NCBI GEO (https://www.ncbi.nlm.nih.gov/geo/) GSE103027 and GSE105120. The corresponding metadata is available in Supplementary Table 1. All other datasets are available from the corresponding author upon reasonable request.
