# Supplementary material for: Host-microbe multi-omics and succinotype profiling have prognostic value for future relapse in patients with inflammatory bowel disease
Source: Gut Microbes. 2025 Jan 15;17(1):2450207. doi: 10.1080/19490976.2025.2450207 (PMC11740686; doi:10.1080/19490976.2025.2450207)
Supplement: Supplemental Material [file KGMI_A_2450207_SM4830.zip › KGMI_A_2450207 supplement/Supplementary_Materials_Gut_Microbes_accepted_ref_list_incl.docx]

**Supplementary Materials:**

Supplementary tables 1-13 are provided in the submitted excel file.

**Supplementary Figure 1:** Read summary of 16S amplicon sequencing datasets during the steps of the DADA2 pipeline

**
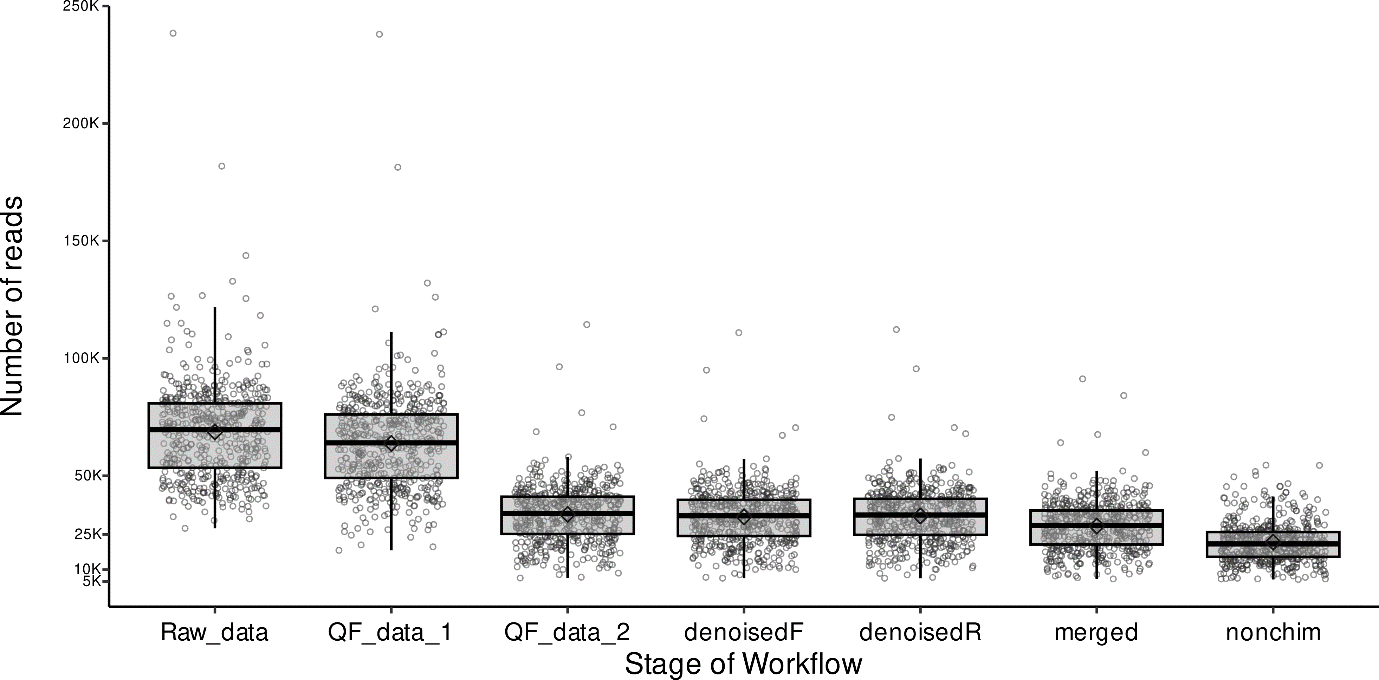
**

**Supplementary Figure 2:** Comparison of microbiota composition and diversity between patients with CD who had reported ileal involvement and those that did not. A-B) Principal component analysis (PCA) based on Aitchison distances grouped by ileal involvement status and inflammation status with analysis repeated for 16S gDNA and cDNA datasets, respectively. C-D) Comparison of Shannon alpha diversity between ileal involvement (Yes) and no ileal involvement (No) groups for gDNA and cDNA datasets respectively.

**
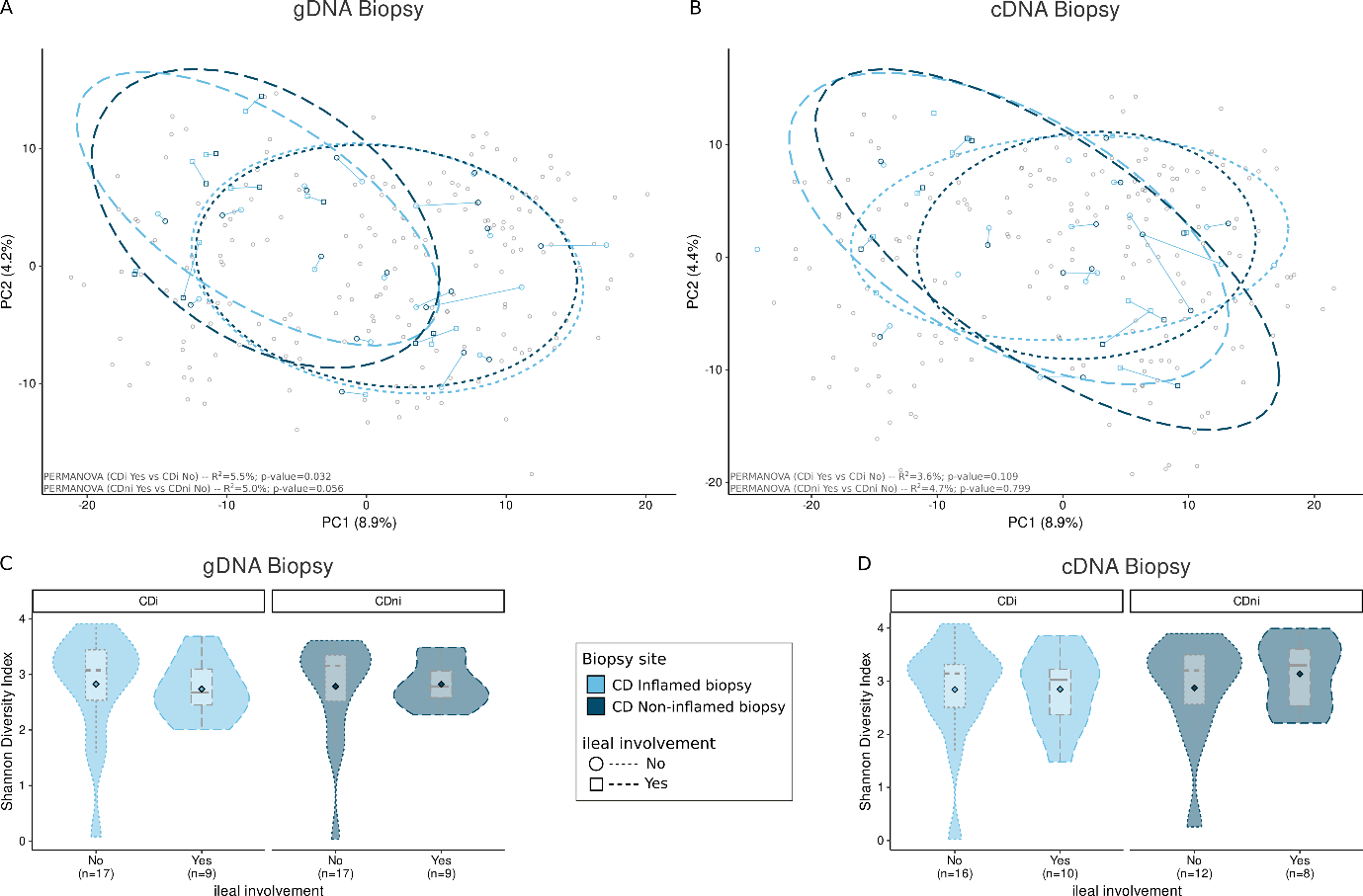
**

**Supplementary Figure 3:** Boxplots of CLR transformed abundances of A) unclassified *Coprococcus,* B) unclassified *Subdoligranulum* and C) *Blautia* *obeum*, split by disease type and inflammations status for both 16S gDNA and 16S cDNA datasets. P-values presented are those generated by ALDEx2 when conducted differential abundance analysis after adjusting for multiple testing. *q < 0.05, **q<0.01, ***q<0.001.

**
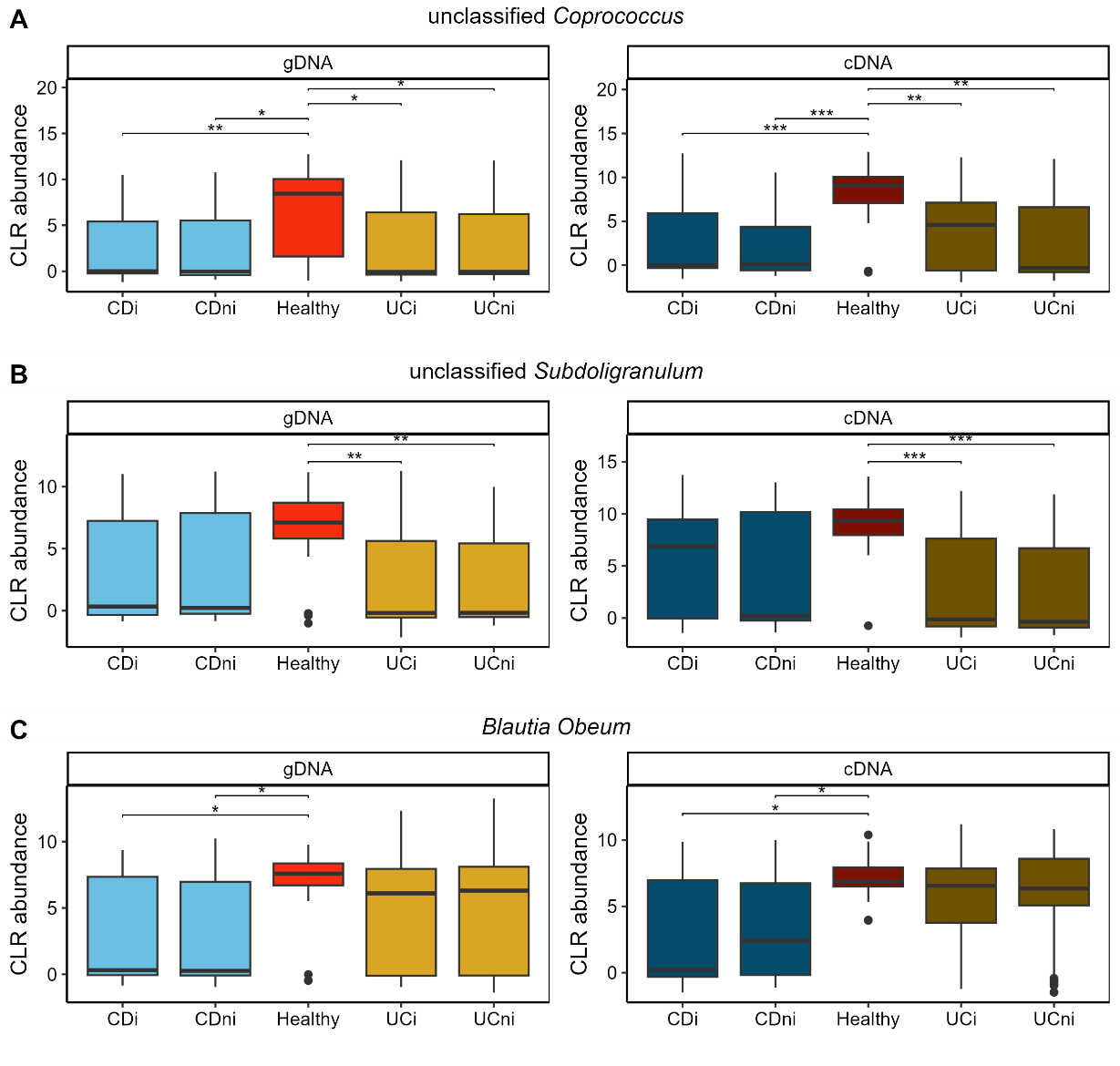
**

**Supplementary Figure 4:** Volcano plots of differential abundance analysis comparing relapse and remission subjects with CD. The analysis was repeated for A-B) gDNA and C-D) cDNA for both inflamed and non-inflamed biopsy samples.

**
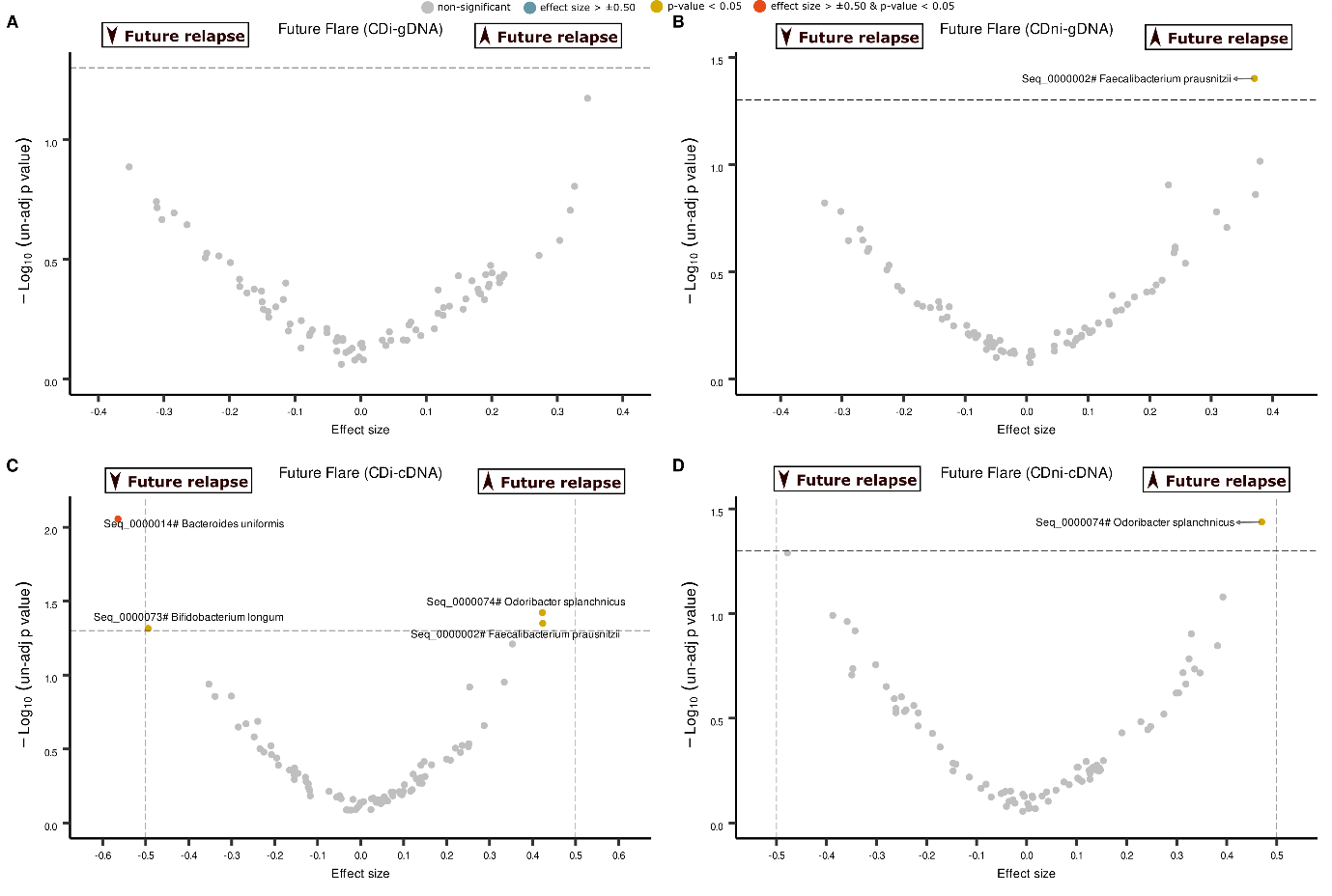
**

**Supplementary Figure 5:** Volcano plots of differential abundance analysis comparing relapse and remission subjects with UC. The analysis was repeated for A-B) gDNA and C-D) cDNA for both inflamed and non-inflamed biopsy samples.

**
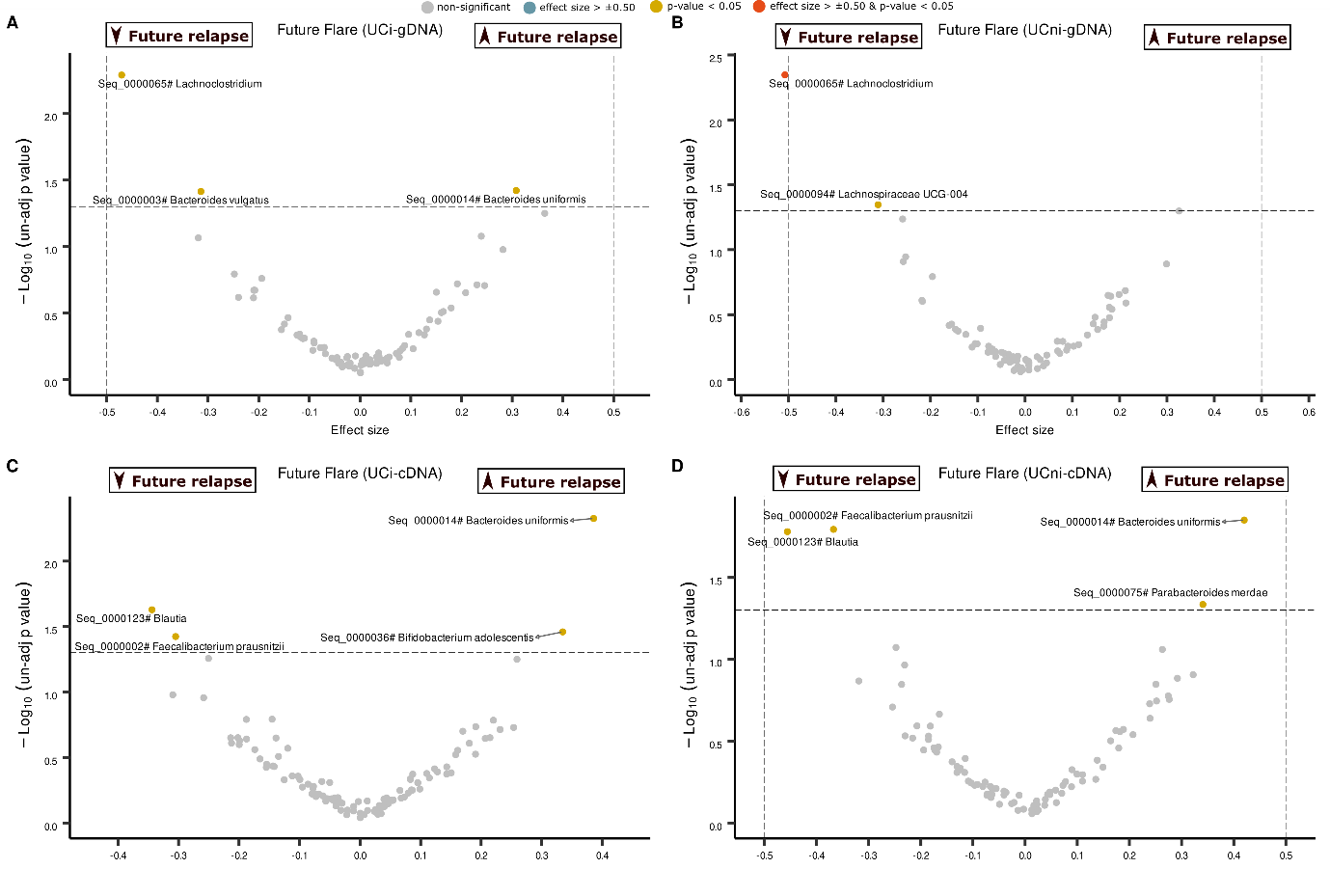
**

**Supplementary Figure 6:** Pathway Enrichment Analysis based on DEGs in A-B) CDi and C-D) UCi samples when using the KEGG and PID database, respectively.

**
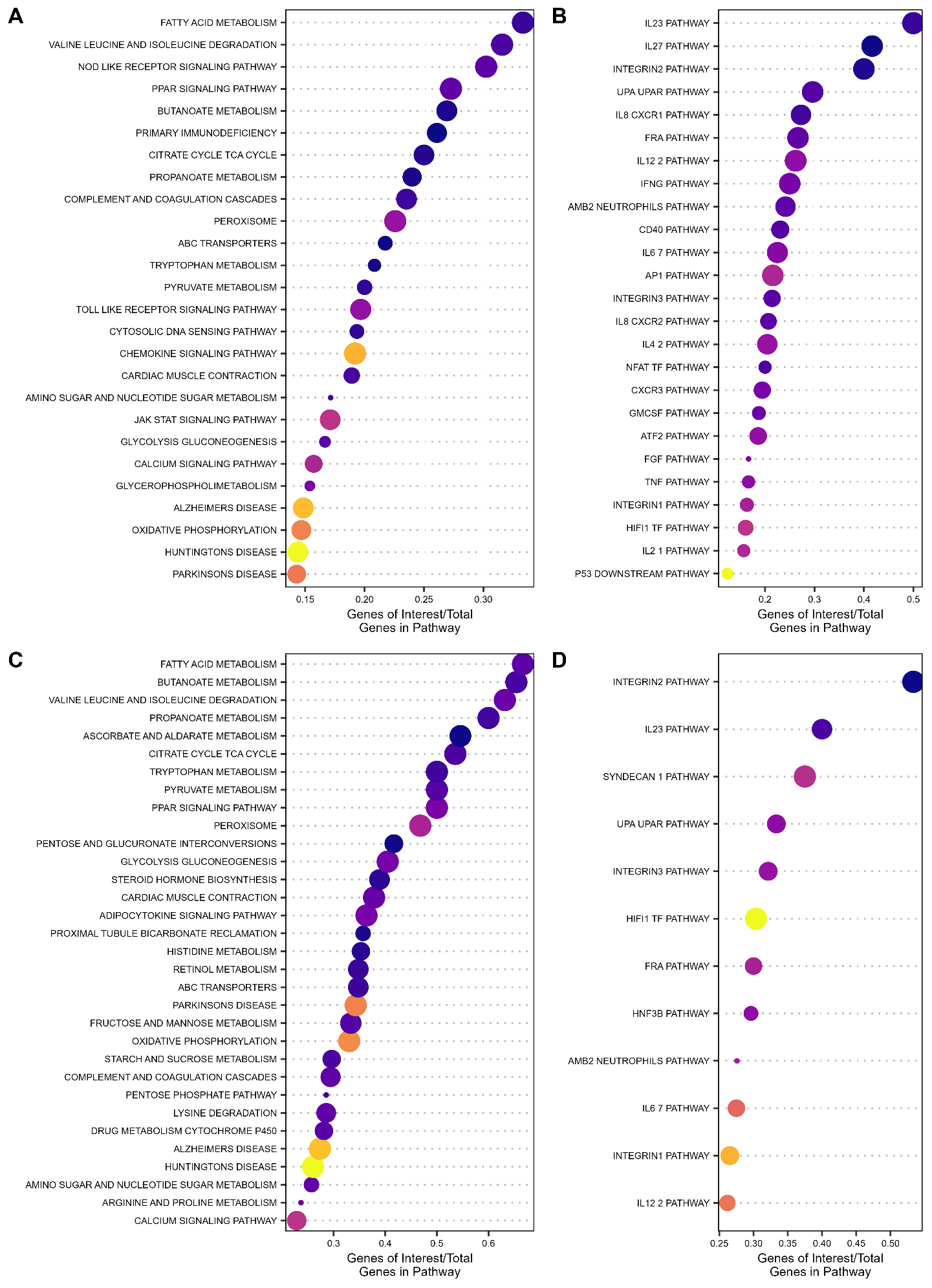
**

**Supplementary Figure 7:** Gene Expression vs Methylation for *AGAP1* gene. A) Boxplots of the methylation of CpG sites associated with the *AGAP1* gene in both inflamed and non-inflamed CD samples. B) Boxplots of the corresponding gene expression in inflamed and non-inflamed CD samples.

**
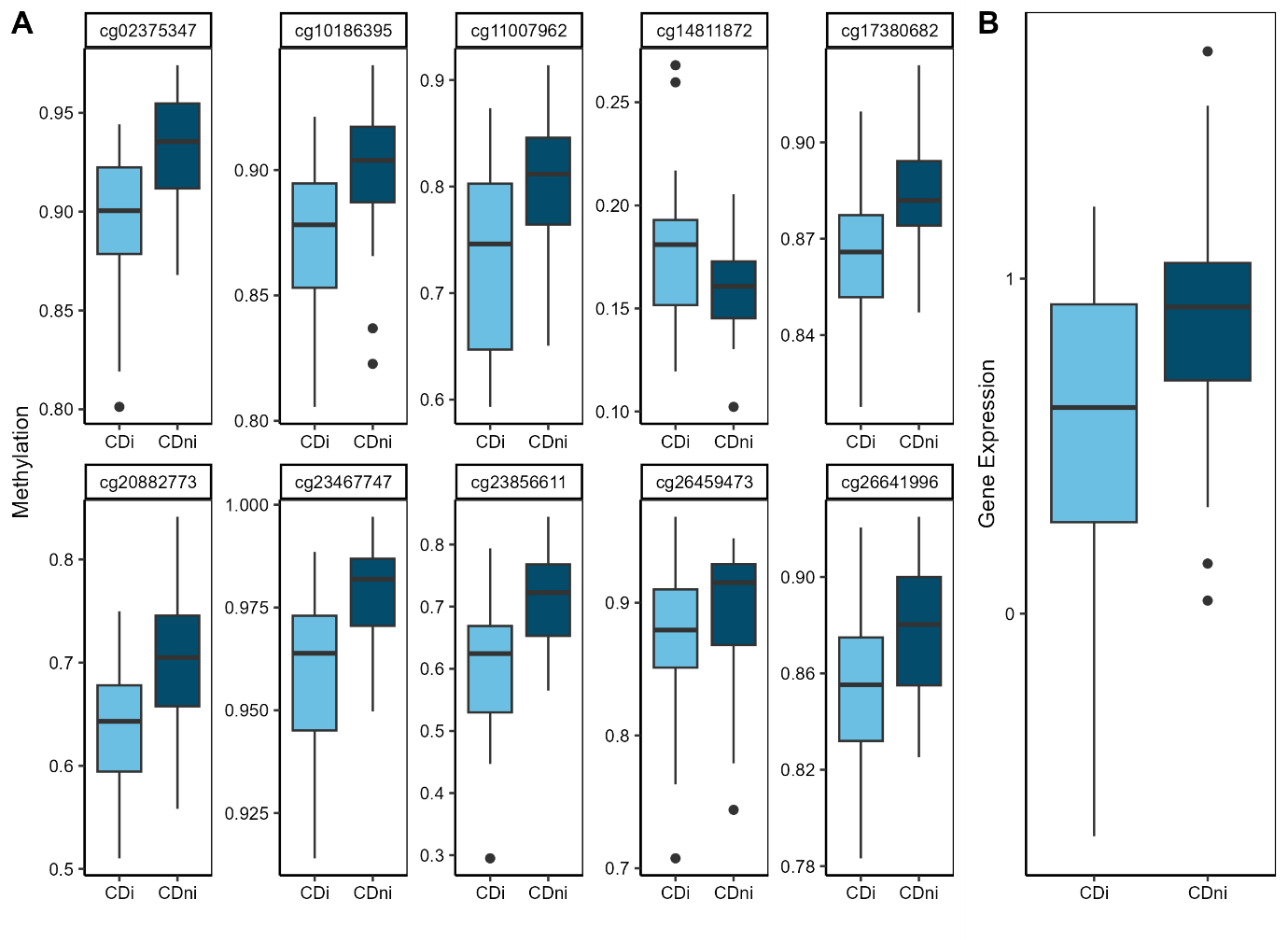
**

**Supplementary Figure 8:** Gene Expression vs Methylation for *PTPRN2* gene. A) Boxplots of the methylation of CpG sites associated with the *PTPRN2* gene in both inflamed and non-inflamed CD samples. B) Boxplot of the corresponding gene expression in inflamed and non-inflamed CD samples.

**
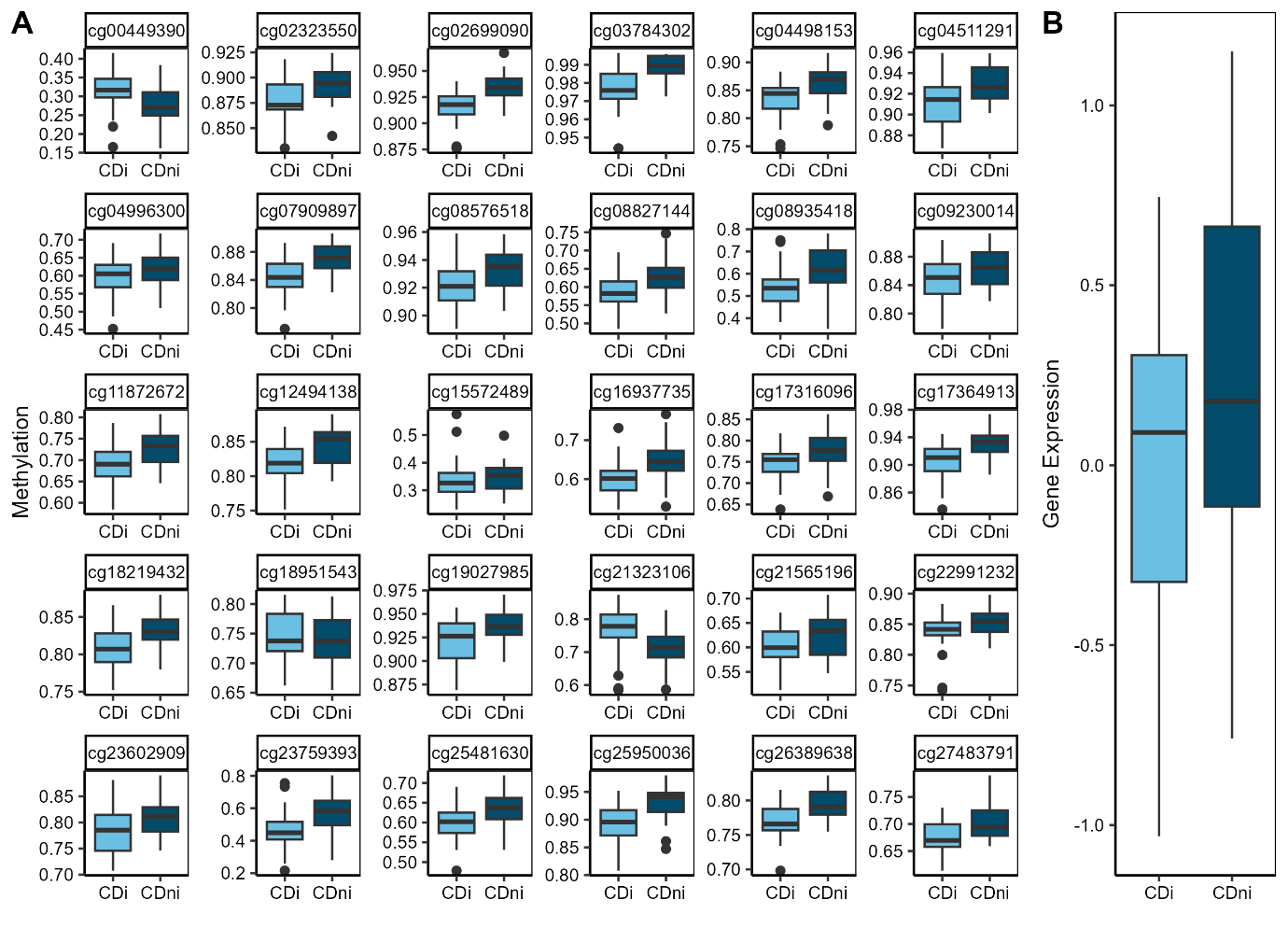
**

**Supplementary Figure 9:** Correlation plots highlighting associations between genes from the Integrin beta-1 pathways and the A) active and B) standing abundances of the *Parasutterella* genus.

**
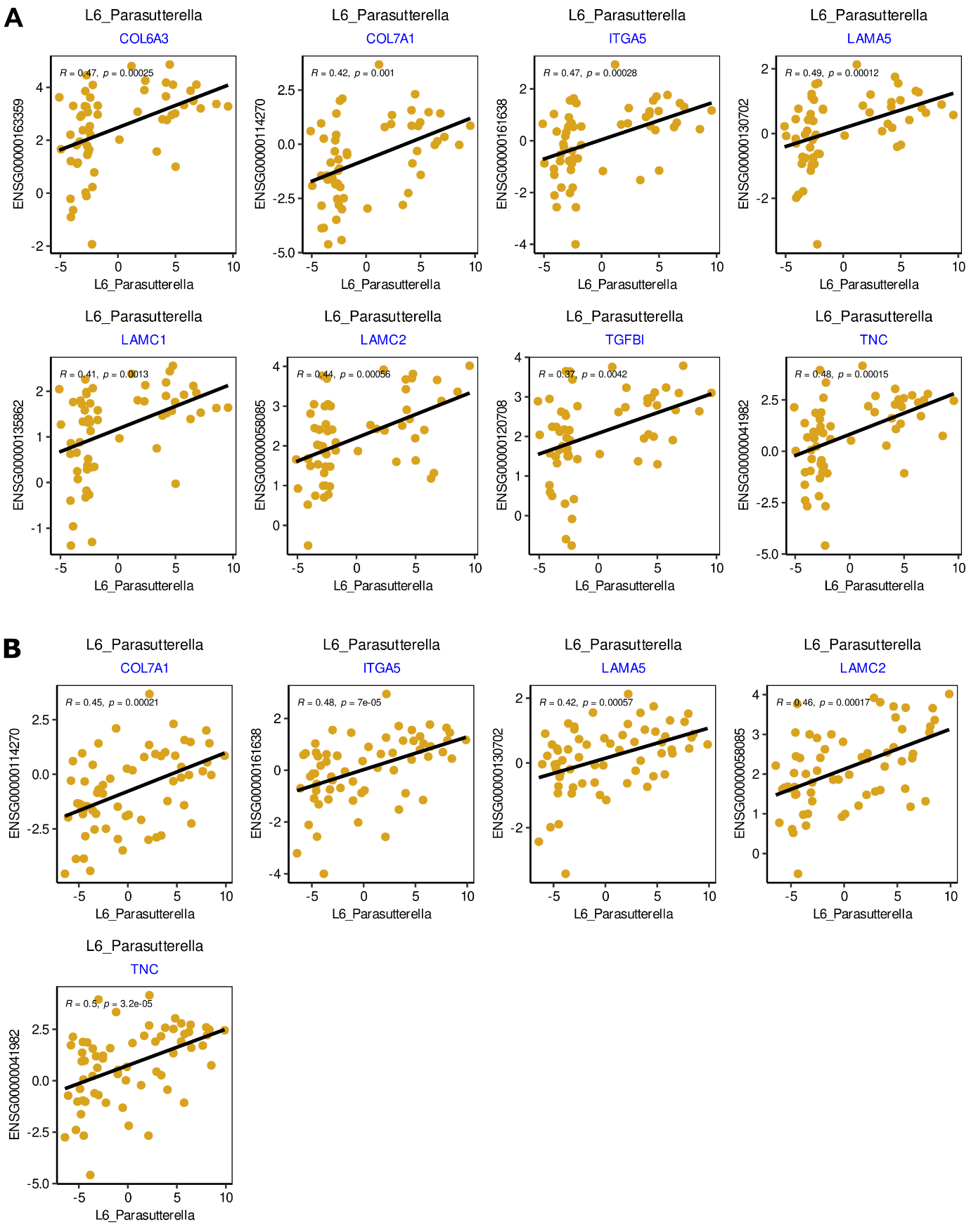
**

**Supplementary Figure 10:** Dot plots of pathways enriched based on significant associations between microbes and methylation of promoter regions. A) Pathways enriched based on promoters found to be associated with the genus *Lachnospiraceae UCG-004*. B) Pathways enriched based on the methylation of promoters found to be associated with a *Lachnospiraceae CAG-56* RSV

**
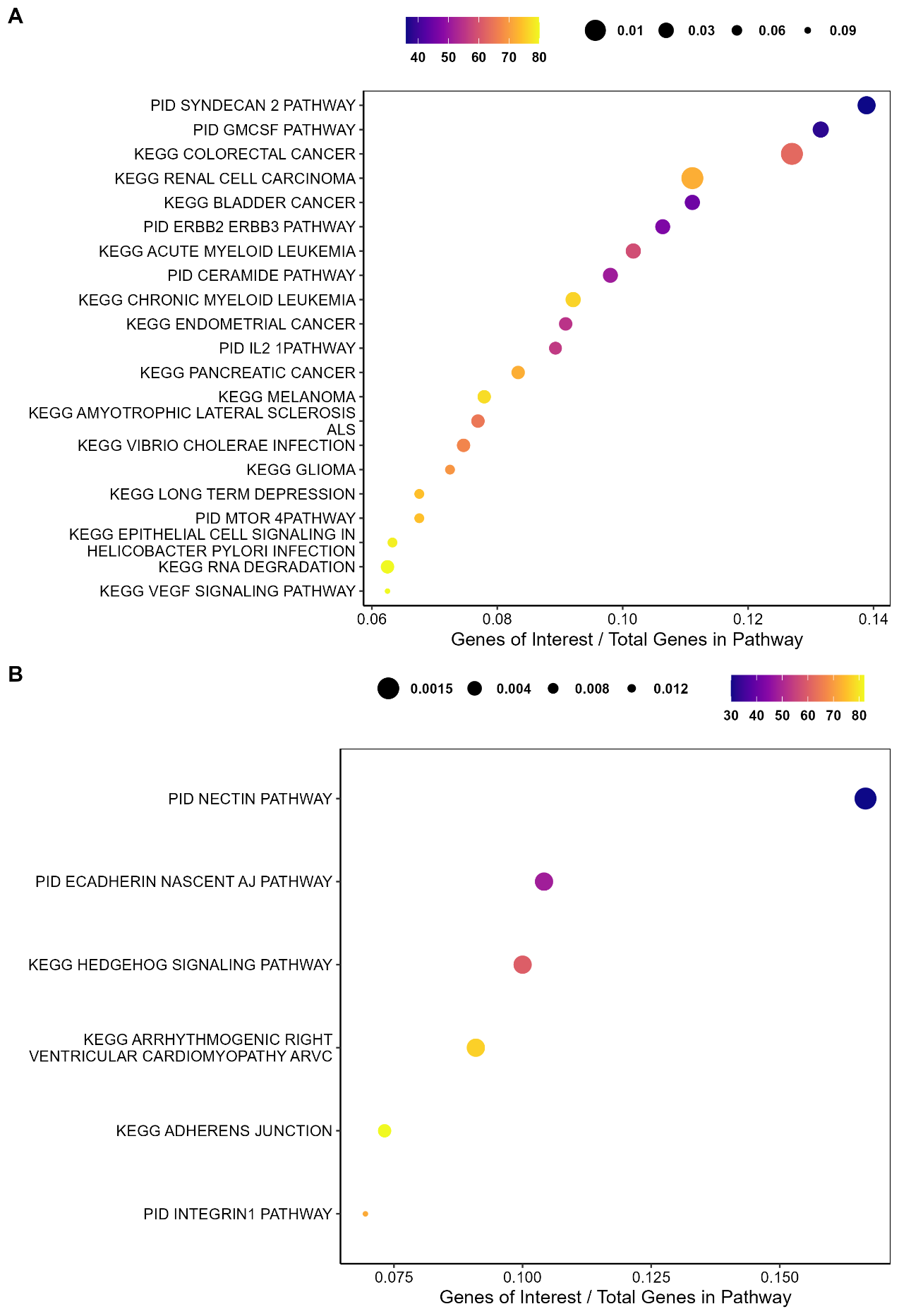
**

**Supplementary Figure 11:** Upset plots outlining XGBoost model performances for the top five models when predicting relapse in IBD patients (CD + UC), where models were trained on inflamed, non-inflamed and paired samples, respectively (left-right). Performance is measured using the AUC metric

**
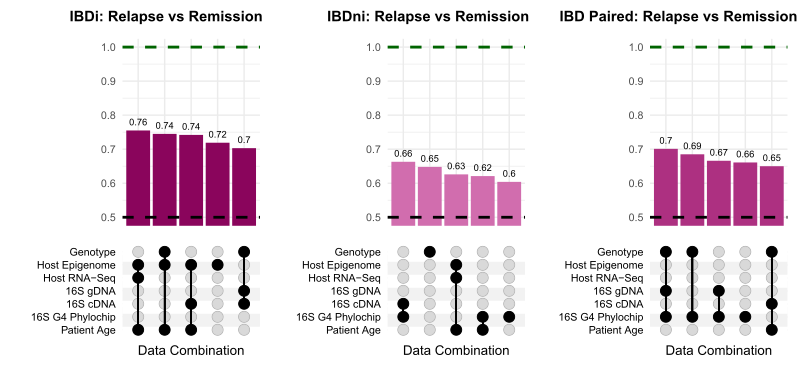
**

**Supplementary Information 1: Test vs Validation Performance of top ML models.**

Given the complex and heterogeneous nature of multi-omics datasets and that no corresponding external validation set was available, we conducted a nested cross validation to train and assess the performance of our models. This allowed us the benefit of assessing the model’s performance on test, validation and training splits which are commonly used in ML model building pipelines. In ML, a training set is used to fit the model, whereas a validation set is used to reduce the error of the model by fine-tuning hyperparameters or performing training early stopping or model checkpoints. The test set is finally used to estimate the model error or performance. The performance on the validation set is a good indicator of the best achievable performance on potential external datasets. By comparing model performance on the validation set and the held-out test set, it is thus possible to get an idea of which models may generalize better to new and independent data and to assess the stability of the model itself.

Furthermore, the problem known as “winner’s curse”, commonly considered during hypothesis testing in statistics^1^, can also occur in ML analysis when multiple models are compared. It often happens when the test set is used to not only to estimate the error, but also to select the winning model (or feature set), thus the process that aimed to estimate the performance is now used to improve it. By considering both the validation and test set performance, we can attempt to minimize this winner’s curse by highlighting the models with both high test and validation performance. Additionally, the gap between validation and test performances is indicative of algorithmic stability of the chosen methodology. That is, if the estimated performance (test data score) is close to the expectations (validation data score) then the created system is stable and reliable. However, when the gap is large then the part of the system that is changed (a feature set) brings a certain distribution shift that affects the performance.

Using the predictions from the inner loop of the nested cross-validation, which were extracted when building the models of our ensemble, it was possible to obtain an estimation of the validation performance of our approach (Supplementary Figure 12). For this analysis, we focused on those models that showed high test performance when classifying relapse and remission groups. This included the top five models trained on inflamed sample data for patients with CD and those top five models trained on inflamed or paired data from patients with UC as outlined in Figure 6. For the models with the highest test AUCs in each scenario, we observed only small differences between test and validation performance (Difference: 0.001-0.06; Supplementary Figure 12). Furthermore, the models with the highest test performances were also among those with the highest validation performance, indicating our top models appear stable between test and validation sets. However, we did observe for some models that as the test performance decreased the gap between test and validation increased, indicating such models may be less reliable for this particular problem. This however would need to be confirmed on future multi-omics datasets from patients with IBD.

**Supplementary Figure 12:** Comparison of test and validation performance of top 5 highest performing models when predicting relapse based on A) CDi, B) UCi and C) UC paired samples respectively.

**
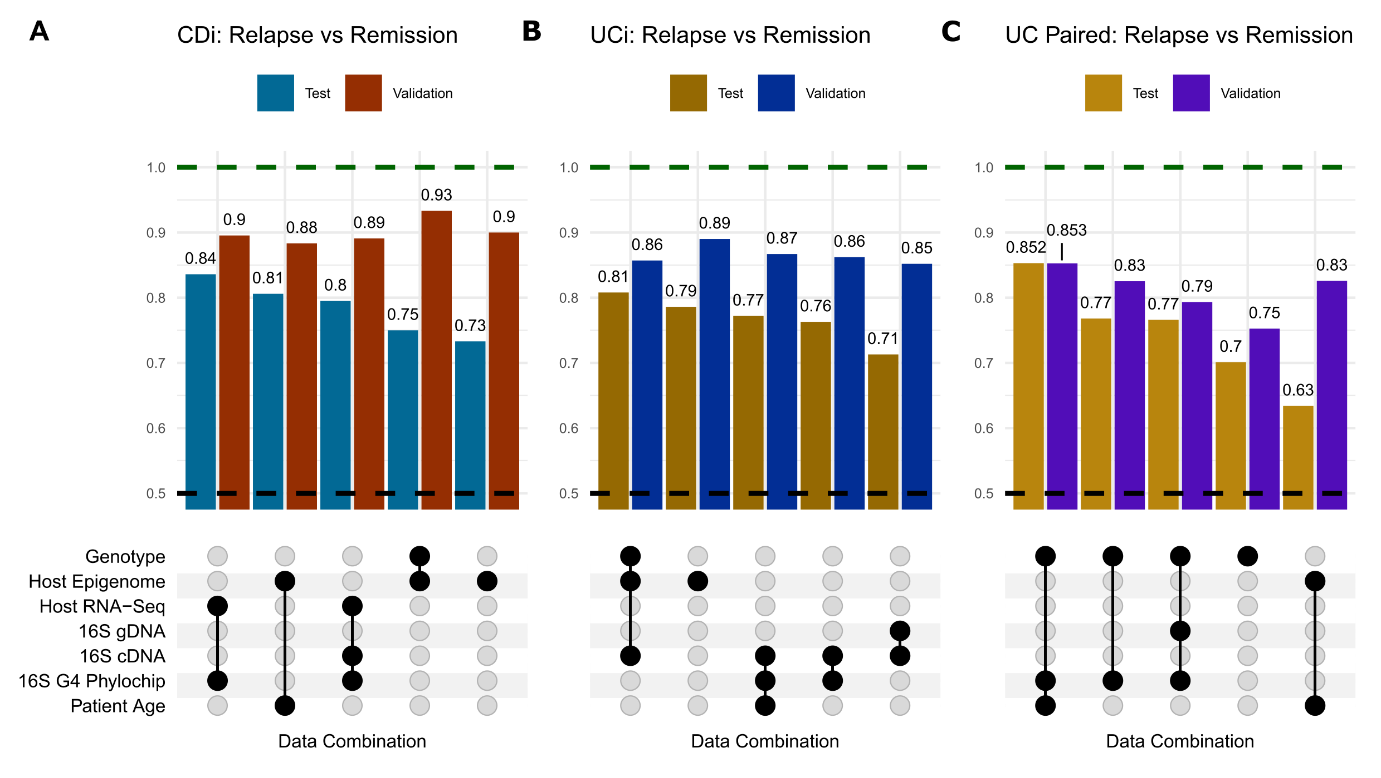
**

**Supplementary Figure 13:** Flowchart of the Machine Learning pipeline used to train models.

**
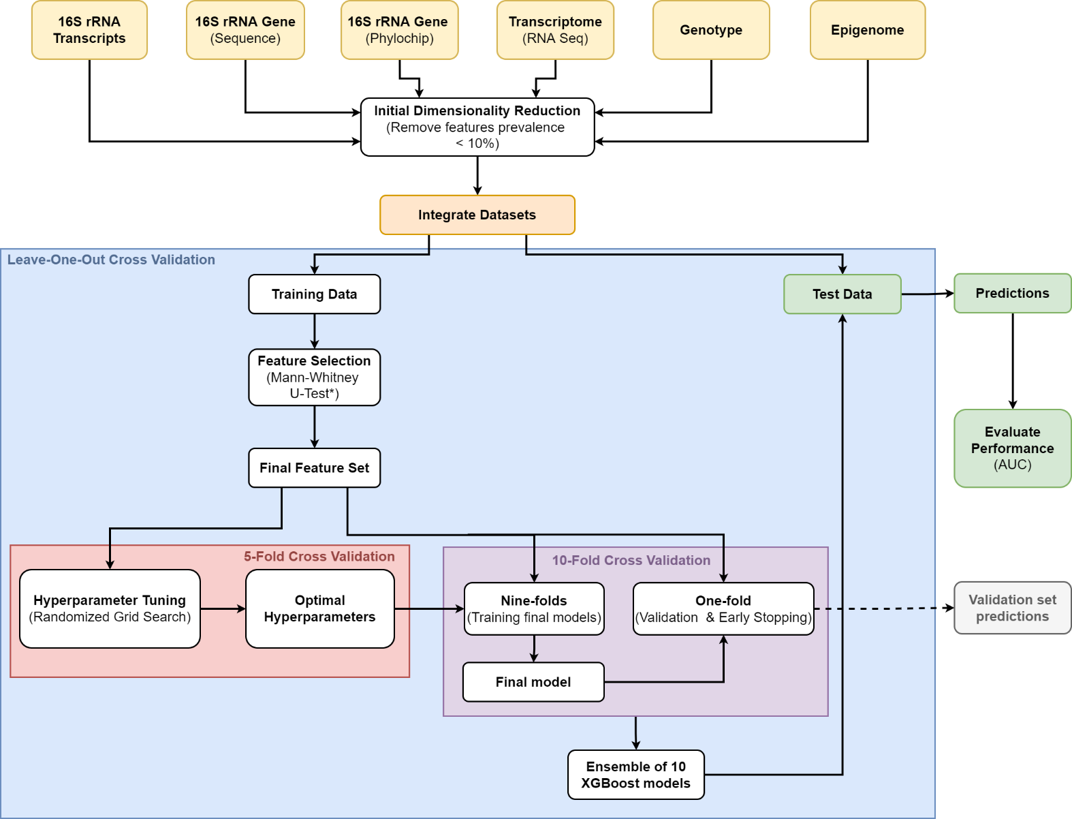
**

**References**

1. Jensen DD, Cohen PR. Multiple comparisons in induction algorithms. *Mach Learn* 2000; 38(3):309–338. doi: 10.1023/A:1007631014630.
